# *CDKN3* mRNA as a Biomarker for Survival and Therapeutic Target in Cervical Cancer

**DOI:** 10.1371/journal.pone.0137397

**Published:** 2015-09-15

**Authors:** Eira Valeria Barrón, Edgar Roman-Bassaure, Ana Laura Sánchez-Sandoval, Ana María Espinosa, Mariano Guardado-Estrada, Ingrid Medina, Eligia Juárez, Ana Alfaro, Miriam Bermúdez, Rubén Zamora, Carlos García-Ruiz, Juan Carlos Gomora, Susana Kofman, E. Martha Pérez-Armendariz, Jaime Berumen

**Affiliations:** 1 Unidad de Medicina Genómica, Facultad de Medicina, Universidad Nacional Autónoma de México/ Hospital General de México, México City, México; 2 Departamento de Medicina Experimental, Facultad de Medicina, Universidad Nacional Autónoma de México, México City, México; 3 Servicio de Oncología, Hospital General de México, México City, México; 4 Departamento de Neuropatología Molecular, División de Neurociencias, Instituto de Fisiología Celular, Universidad Nacional Autónoma de México, México City, México; 5 Departamento de Inmunología, Instituto de Investigaciones Biomédicas, Universidad Nacional Autónoma de México, México City, México; 6 Laboratorio de Biología Molecular, Asociación para Evitar la Ceguera en México Hospital Dr. Luis Sánchez-Bulnes, México City, México; 7 Servicio de Genética, Hospital General de México/Facultad de Medicina, Universidad Nacional Autónoma de México, México City, México; National Institute of Health—National Cancer Institute, UNITED STATES

## Abstract

The cyclin-dependent kinase inhibitor 3 (*CDKN3*) gene, involved in mitosis, is upregulated in cervical cancer (CC). We investigated *CDKN3* mRNA as a survival biomarker and potential therapeutic target for CC. *CDKN3* mRNA was measured in 134 CC and 25 controls by quantitative PCR. A 5-year survival study was conducted in 121 of these CC patients. Furthermore, *CDKN3*-specific siRNAs were used to investigate whether *CDKN3* is involved in proliferation, migration, and invasion in CC-derived cell lines (SiHa, CaSki, HeLa). *CDKN3* mRNA was on average 6.4-fold higher in tumors than in controls (p = 8 x 10^−6^, Mann-Whitney). A total of 68.2% of CC patients over expressing *CDKN3* gene (fold change ≥ 17) died within two years of diagnosis, independent of the clinical stage and HPV type (Hazard Ratio = 5.0, 95% CI: 2.5–10, p = 3.3 x 10^−6^, Cox proportional-hazards regression). In contrast, only 19.2% of the patients with lower *CDKN3* expression died in the same period. In vitro inactivation of *CDKN3* decreased cell proliferation on average 67%, although it had no effect on cell migration and invasion. *CDKN3* mRNA may be a good survival biomarker and potential therapeutic target in CC.

## Introduction

Cervical cancer (CC) is the fourth most common cancer in women worldwide [1]. Each year 530,000 new cases are reported, making it the leading cause of death by cancer in women from developing countries [2, 3]. Human papillomavirus (HPV) is present in almost 100% of CCs patients and is considered the main cause for development of CC. Worldwide, HPV 16 is the most frequently detected viral type in CC, found in approximately 50% of cases, followed by HPV 18 (15%) [4, 5]. Vaccines currently used are very effective in preventing infections by HPV types 16 and 18. They also prevent development of high-grade cervical intraepithelial neoplasia associated with these viruses [6, 7]. However, since these vaccines offer protection against only some viruses and the duration of the immune response is unknown, it is recommended that vaccinated women continue to be enrolled in early detection programs for CC [8, 9]. In many countries, these vaccines have been incorporated into vaccination programs for girls aged 9–12 years [10, 11]. Despite vaccine implementation, the natural history of the disease indicates that an impact on cervical cancer incidence will not be seen for decades. [10, 12]. Furthermore, according to the distribution of HPV types 16 and 18 among various age groups, about half of the women over 50 years with CC would not be protected by such preventive vaccines [13]. Therefore, it is necessary to improve procedures for early detection and treatment of CC.

Current treatment for CC includes surgery, chemotherapy, radiation therapy, or a combination of these therapies, depending on the clinical stage of the disease. Although the success of these conventional methods depends on the clinical stage of the disease, in general, patient survival decreases with the stage of the disease [14]. While there are targeted therapies for many types of cancer [15], there are no specific targeted therapies against CC. Mutated oncoproteins, especially protein kinases, are the targets of most specific-target cancer drugs [16]. In addition, some specific drugs target normal proteins upregulated in tumors, such as HER2/neu in breast cancer [17, 18]. Identification of upregulated molecular targets in CC, which are essential for tumor growth and absent in healthy cervix, is the first step towards development of specific target drugs for treatment of CC.

The inhibition of mitosis is a well-known strategy to combat cancer [19, 20]. Drugs that inhibit the process of cell division are usually effective as anticancer agents. The taxanes and vinca alkaloids, which inhibit the formation of the mitotic spindle, are well-known examples of these drugs. However, their effectiveness is limited because they also affect the microtubule network of cells that do not divide affecting endothelial functions and producing neurotoxic effects.

In a previous work, we demonstrated that the gene "cyclin-dependent kinase inhibitor 3" (*CDKN3*), which is involved in mitosis, is upregulated in CC compared to that in normal cervical epithelium. Furthermore, in a preliminary 3.5-year survival analysis, including a small sample of CC patients (n = 42) positive for HPV16, the high expression levels of *CDKN3* have been found associated with a shorter patient survival [21]. These data suggest that *CDKN3* may be involved in tumor progression, at least in CC positive for HPV16. It is not known whether the over expression of *CDKN3* is also associated with a survival decrease in CC patients positive for HPVs other than HPV16.

To demonstrate conclusively whether the overexpression of *CDKN3* is associated with reduced survival time in CC patients, a 5-year survival analysis was conducted in a larger sample (n = 121). This included a group of patients positive for HPVs other than HPV16 and a larger group of HPV16-positive patients. Furthermore, to investigate whether *CDKN3* is involved in the carcinogenesis process, we analyzed the effect of the inhibition of *CDKN3* gene expression on the proliferation, migration, and invasion of cell lines derived from HPV16-positive (SiHa and CaSki) and HPV18-positive (HeLa) CCs.

## Methods

### Patient selection, study design, and endpoints

The study subjects included 134 patients diagnosed with CC at the Department of Oncology and 25 women with normal cervical epithelium (experimental controls) evaluated at the Department of Obstetrics and Gynecology at the Hospital General de México in Mexico City. The CC samples were a subset selected from a total of 462 patients with CC who were recruited with the following inclusion criteria: clinical diagnosis of invasive CC at the Oncology Department, no previous treatments, and born and resident in Mexico with a Mexican ancestry going back two generations. Patients who fulfilled the inclusion criteria were subsequently recruited during the periods from November 2003 through April 2005 and January 2006 through July 2007, and represented about 80% of the patients diagnosed with CC during this period. The selection criteria for the CC subset were based on the availability of high-quality RNA extracted from fresh tumor biopsy with more than 70% tumor cells in the morphological analysis and the viral type positivity of tumors. Based on the quality of RNA, most cases (88%) were selected during the second recruitment period. CCs included 90 samples positive for HPV16 (42 previously reported [21], which were updated with the 5-years survival data, and 48 new samples analyzed for this report) and 44 positive for other HPV types: 18, 31, 33, 35, 45, 51, 52, 53, 58, 59, and 68. The women included in this report were comparable to those excluded based on age (mean = 50 years), race/ethnicity, histology, and tumor stage. The frequency of HPV types in this subset was not comparable to the frequency in the whole sample [13], since a higher proportion of HPV16-positive tumors were selected. The tumors of CC patients were staged according to the Fédération Internationale de Gynécologie Obstétrique (FIGO) [22]. Two biopsy samples were taken from the tumors during colposcopy examinations. One sample was divided into two equal parts: one part was fixed in buffered formalin for morphological analysis and the other part, together with the second biopsy sample, was snap-frozen on dry ice and stored at −80°C until analysis. Control cervical specimens were obtained from patients undergoing hysterectomy due to myomatosis. The race and mean age (49 years) of these patients was similar to that of the patients with CC. They were previously diagnosed with a normal cervix by cytology and colposcopy. Immediately after receiving a cervical fragment from the operating room, we dissected the exocervical epitheliums under a stereoscopic microscope to avoid stromal cells. Only four samples (16%) were positive for HPV but the signals were weak. The primary endpoint was the 5-year overall survival. The study protocol was approved by the Scientific and Ethics Committees of the Hospital General de Mexico (approval number DIC/03/311/04/051) and informed written consent was obtained from all participants prior to their inclusion.

### DNA and RNA isolation

DNA was purified from biopsy specimens using a PureLink Genomic DNA Kit (Invitrogen, Carlsbad, CA, USA) and maintained at −20°C until analysis. Total RNA was isolated from half of the divided biopsy using TRIzol reagent (Invitrogen) according to the manufacturer’s protocol. The quality of the RNA was confirmed with agarose gel electrophoresis, as demonstrated by the presence of intact ribosomal RNA, with the 28S band twice as intense as the 18S band.

### Detection and HPV typing

The HPV detection was performed by PCR using universal primers located in HPV L1 genes *MY09*/*MY11*, *GP5+*/*6+*, and *L1C1*, as described previously [23–25]. The *HBB* gene was used as an internal control to assess the quality of the DNA. The HPV types were identified by sequencing the amplified bands using the fluorescent cycle-sequencing method (BigDye Terminator Ready Reaction Kit; Applied Biosystems, Carlsbad, CA, USA). Sequence analysis was performed using an ABI PRISM 3130xl Genetic Analyzer system (Applied Biosystems). Each band sequenced was analyzed with the FASTA sequence similarity tool [13, 26]. The average identity percentage of HPV types detected was 98.7% when compared to the reference sequences.

### Measurement of *CDKN3* gene expression using quantitative real-time reverse transcription polymerase chain reaction (qRT-PCR)

Reverse transcription of total RNA was performed using the High-Capacity cDNA Archive kit (Applied Biosystems) in a total volume of 20 μL. The mix included 2 μg of RNA, 2 μL of 10× RT buffer, 0.8 μL of 100 mM dNTPs, 2 μL of 10× RT Random Primers, 1 μL of MultiScribe^TM^ reverse transcriptase (5 U/μL), and 1 μL of RNase inhibitor (2 U/μL). Reactions were incubated at 37°C for 120 min, and then stored at −20°C. *CDKN3* gene expression was measured in 134 CC and 25 healthy cervical epithelium control samples by reverse transcription-quantitative PCR (RT-qPCR) using TaqMan probes. *GAPDH* was used as internal control. TaqMan gene expression assays were used (*CDKN3*, Hs00193192_m1; *GAPDH*, Hs02758991_g1; Applied Biosystems). The experiments were run in triplicate in a final volume of 20 μL, including 200 ng of cDNA template, 10 μL of 2× TaqMan Universal PCR Master Mix (Applied Biosystems), 1 μL of 20× TaqMan Gene Expression Assay, and 7 μL of RNase-free water. The cycling program was run in a Rotor-Gene (Corbett Research, Sydney, Australia), which was set as follows: an initial PCR activation step at 50°C for 2 min followed by 95°C for 10 min, then 40 cycles of melting at 95°C for 15 s and annealing/extension at 60°C for 1 min. Measurement of gene expression was based on relative standard curves constructed from a 10-fold serially diluted pool of CC cDNAs ranging from 500 to 0.05 ng. The expression of *CDKN3* gene was normalized in each tumor and control sample to the intensity of the internal reference (*GAPDH*) using a previously described method [21]. The normalized intensity values were measured in ng/μL. A normality test (Shapiro-Wilk) was carried out to test for a normal distribution of gene expression data. The fold-change expression was calculated by dividing the median normalized intensity of each tumor sample by the median normalized intensity of the control samples. The statistical significance between the medians of tumors and controls was calculated with the Mann–Whitney (MW) non-parametric test.

### Identification of *CDKN3* RNA variants using RT-PCR and DNA sequencing

*CDKN3* RNA variants were determined in the cDNA of 45 tumors, 22 normal cervical epithelium, and three cell lines (CaSki, Hela, and SiHA) using RT-PCR. The primers used for the RT-PCR were previously described [27] or designed according to published variants sequences [28] (S1 Table). The PCR products were analyzed using agarose gel electrophoresis and sequenced using the fluorescent cycle-sequencing method (BigDye Terminator Ready Reaction Kit; Applied Biosystems). Sequence analysis was performed using an ABI PRISM 3130xl Genetic Analyzer system (Applied Biosystems). Sequences were analyzed with the FASTA sequence similarity tool [13, 26], SeqScape software (Applied Biosystems), and ClustalW2 alignment tool (http://www.ebi.ac.uk/Tools/msa/clustalw2/).

### Survival analysis

According to FIGO staging patients with cervical cancer received individualized treatment based on the treatment guidelines for cervical cancer of the American Cancer Society (S2 Table). After the treatment was completed, each patient was clinically evaluated every 1, 3 or 6 months by an experienced oncologist. Clinical data of the follow-up study was obtained from the patients’ medical record. Also, a social worker performed phone calls and home visits to the patients every 6 months during the study. Only 121 of 134 patients explored with qRT-PCR were included in the follow-up study, and included 83 samples positive for HPV16 and 38 samples positive for other HPV types, including HPV18, 31, 33, 35, 45, 51, 52, 53, 58, 59 and 68. Survival analysis was performed on all patients who received treatment and were followed up at least 10 months. Those marked with an asterisk in the S2 Table did not receive treatment (n = 8) or were lost during the follow up in the first 10 months (n = 5). The median following time of the 121 patients was 64 months after initial diagnosis. Status alive was registered at the last follow up, death was caused by primary tumor of cervical cancer, except the case labeled with a double asterisk in S2 Table (R221), and unknown cases were referred to those patients we lost track of their status before 60 months of follow-up (R251, R278, R289 and R379). These lost cases and the patient deceased from causes other than cervical cancer (R221) were designated as censored. Censored and deceased patients were followed up for the number of months indicated in S2 Table. Patients were considered lost when did not attend to medical appointments for disease control, were not found at home visits or did not answer phone calls. The cause of death of all patients during the follow up was confirmed by the medical record and the death certificate. The association of FIGO, HPV type and *CDKN3* expression with survival was investigated by survival analysis (see below in the section of statistical analysis).

### Cell lines

The CC cell lines positive for HPV16 (CaSki, SiHa) and HPV18 (HeLa) were provided by Dr. J. C. Gomora from the Department of Biophysics of IFC-UNAM, México City. The cells were cultured with Dulbecco's Modified Eagle's Medium (Gibco® DMEM Cat: 12100–038, Life Technologies, Grand Island, NY) supplemented with fetal bovine serum (Gibco® Cat: 16000–044, Life Technologies) and antibiotic-antimycotic solution (Gibco® Cat: 15240–062, Life Technologies) at 37°C in a 5% CO_2_ incubator. Cell lines were authenticated by short tandem repeat profiling prior the experiments (data not shown).

### Transfection of siRNAs

The experiments were repeated two times in triplicate. A total of 2 × 10^5^ cells were seeded in each well of a twelve-well plate and grown to 60–80% of confluency prior to transfection. Cells were transfected with a mixture of three specific siRNAs against *CDKN3* (*CDKN3* siRNA, sc-43877) or control siRNAs which contain three scrambled sequences that will not lead to the specific degradation of any know cellular mRNA (control siRNA-A, sc-37007), using the siRNA Transfection Reagent (sc-29528) according to the manufacturer’s instruction (all regents from Santa Cruz Biotechnology, Inc., CA). Different concentrations of siRNAs were explored (80, 100, and 120 nM) to investigate the appropriate concentration to inhibit *CDKN3* mRNA in cell lines, incubating the cells 48 h after transfection. The *CDKN3* gene expression level was measured by RT-qPCR as described above.

### Immunofluorescence staining

CDKN3 protein expression was determined in the three cell lines transfected with the specific *CDKN3* or scrambled siRNAs by immunofluorescence. Transfected cells were grown directly on coverslips deposited in the plate wells. Cells cultured 96 h after transfection were fixed in absolute ethanol for 20 min at -20°C. Then, a solution containing 5% of bovine serum albumin in PBS and 0.05% Triton X-100 for cell permeabilization was added and incubated for 30 min. The primary rabbit polyclonal antibody against CDKN3, obtained from Abcam (Cat. 118702; Abcam Biochemicals, Cambridge, MA), was diluted 1:200 in 5% of bovine serum albumin in PBS and 0.05% Triton X-100. A total volume of 20 μL was added to each coverslip, which were incubated overnight at 4°C in a moist chamber. After washing with PBS, the antigen-antibody complexes were detected by incubating the coverslips with 20 μL of anti-rabbit FITC-conjugated secondary antibody (1:100; ZyMax^TM^, Life Technologies Jackson Lab) 2 hours at room temperature. Cell nuclei were counterstained with DAPI [4', 6-Diamidino-2-Phenylindole, Dihydrochloride] (1:1000; Invitrogen). Assays were performed in triplicate. As we have reported previously [21], in tissue slides of normal cervical epithelium, this CDKN3 detection system, involving primary and secondary antibodies, did not give any signal (data not shown). Similarly, in cell line experiments used to test the specificity of the system, in which the primary antibody was not included, the detection system did not give any signal (S1 Fig). These data indicate that our system is specific for CDKN3 protein detection. Images were viewed at 60× magnification using a confocal Olympus fluorescence microscope (Olympus FV1000, Center Valley, PA), and digital images were acquired with a CCD camera. The green fluorescence intensity was quantified in 140 fields of each experiment using ImageJ software [29].

### Immunohistochemistry (IH)

The protein expression of CDKN3 was determined in 37 CC (22 and 15 positive for HPV16 and other HPVs, respectively) and 21 control samples using immunohistochemistry (IH). Five tissue microarrays (TMA) were built with each containing 10–12 CCs and 4–5 controls. The assay was performed as previously described [21]. In brief, the TMA were deparaffinized in xylene and then rehydrated sequentially with graded concentrations of alcohol. The primary antibody against CDKN3 (sc-475, 1:100) was obtained from Santa Cruz Biotechnology (Santa Cruz, CA, USA). Antigen-antibody complexes were detected using the avidin-biotin peroxidase method, with 3,3′-diaminobenzidine-tetrahydrocloride as a chromogenic substrate (Cat. KO679 LSAB+Sys/HRP; Dako-Cytomation Carpinteria, CA, USA) and the sections were counterstained with hematoxylin. Assays were performed in triplicate. The cellular localization of the immunoreaction was identified, and the intensity was scored 0–4, where 0 indicated no staining and 4 the most intense staining.

### Western blotting

CDKN3 protein expression was determined using western blotting in the three cell lines transfected with the specific CDKN3 or scrambled siRNAs. Proteins were obtained in radioimmunoprecipitation assay (RIPA) lysis reagent (Cat: 89900, Life Technologies). The protein content of each lysate was determined using a Bradford protein assay (Cat: B6916, Sigma-Aldrich, St Louis, MO, USA) with bovine serum albumin (BSA) as a standard. A 50-μg sample of each lysate was resolved on 12% denaturing polyacrylamide gels (with 5% polyacrylamide stacking gel) and transferred electrophoretically onto a nitrocellulose membrane. After blocking with 5% nonfat dry milk in Tris-buffered saline plus Tween (TBST), the membrane was incubated with a rabbit polyclonal antihuman CDKN3 antibody (sc-475 Santa Cruz Biotechnology, Inc.) and a mouse monoclonal antihuman actin antibody (the monoclonal anti-actin antibody was kindly donated by Manuel Hernámdez, Ph.D, CINVESTAV) overnight at 4°C. The membrane was then incubated with horseradish peroxidase (HRP)–conjugated secondary antibodies (GE Healthcare Bio-Sciences) for 1 h at room temperature. After washing with TBST, the immunoreactive proteins were developed using the enhanced chemiluminescence kit (GE Healthcare Bio-Sciences).

### Proliferation assay

The experiments were repeated two times in triplicate. Cells transfected either with the specific siRNAs against *CDKN3* mRNA or control siRNAs were seeded in each well of twenty four-well plates and harvested at 24, 48, 72, and 96 h after transfection. Cell proliferation was measured with a colorimetric method based on the conversion of tetrazolium salts to formazan by dehydrogenase activity in active mitochondria (Vybrant^®^ MTT Cell Proliferation Assay Kit, V-13154, Life Technologies). Briefly, after replacing the culture medium with 400 μL of fresh culture medium (Gibco ® DMEM no phenol red, Life Technologies), 20 μL of MTT (3-(4, 5-dimethylthiazol-2-yl)-2, 5-diphenyltetrazolium) solution were added in each well, including negative controls without cells. The cells were incubated at 37°C for 3 h, then all of the culture medium were removed and replaced with 350μl of dimethyl sulfoxide (DMSO), mixed gently, and incubate at 37°C for 10 min. The plates were read with a spectrophotometer at 560 nm (BioPhotometer Eppendorf). Cell growth follows an exponential trend predicted by the equation y_t_ = y_0 *_ e^rx^, where y_t_ = absorbance at specific time, x = incubation time in hours, y_0_ = a constant absorbance at time = 0, and r = rate growth. Cell doubling time (Td) was calculated according to two different methods: 1) the formula: Td = (t_2_-t_1_) x [log2/(log y_t2_—log y_t1_)], where y_t1_ is the absorbance at t_1_ (24 h), y_t2_ is the absorbance at t_2_ (96 h), and 2) the formula Td = ln(2)/r. The effect on duplication rate was calculated by dividing the doubling time of the transfected cells with specific siRNAs against *CDKN3* by the doubling time of the transfected cells with scrambled siRNAs.

### Invasion and migration assays

The experiments were repeated two times in triplicate. Invasion assay was done in a 24-well transwell chamber (BD BioCoat™ Matrigel™ Invasion Chamber #354480; Becton Dickinson, Franklin Lakes, NJ). Cells suspended in 250 μL of serum-free medium (5.0 x 10^4^ cells) were added to matrigel-coated filters in triplicate wells. In the lower compartment of the chambers 500 μL of bovine-fetal-serum-conditioned medium was used as chemo attractant. After 48 h of incubation at 37°C in a 5% CO_2_ incubator, cells that invaded through the filters were collected from the lower compartment and stained with MTT colorimetric assay described above. The migration assay was conducted with a similar procedure. Cells were loaded on transwell polycarbonate membrane inserts (BD BioCoat™ Control Inserts 24-well, Cat# 354578; Becton Dickinson) in triplicate wells. The plates were incubated for 22 h at 37°C in a 5% CO_2_ incubator. The cells that had migrated to the lower compartment of the chambers were collected and stained with MTT colorimetric assay described above.

### Statistical analysis

The statistical significance of differences in *CDKN3* expression between tumors and controls was calculated with the Mann–Whitney (MW) non-parametric test. Receiver Operator Characteristic (ROC) curve analysis was performed and Youden index was used [21] to select the best cut-off points to distinguish tumors from death and alive patients in the random selected training sets, using the FC expression values of *CDKN3* gene obtained by RT-qPCR. In brief, with the whole sample set, 500 training sets of 60 samples were randomly created. To categorize the *CDKN3* gene expression data quantified by qRT-PCR, ROC analysis was performed in each training set. This analysis was done to set a cut-off for gene expression that represented those values with the highest sensitivity and specificity to differentiate between dead and surviving patients. The whole sample set was then analyzed with the average cut-off, calculated from the values of the 500 training sets. Samples with *CDKN3* gene expression values equal or above the cut-off were set to 1 and those with values below the cut-off were set to 0.

The cumulative overall survival time was calculated by the Kaplan-Meier method and analyzed by the log-rank test. FIGO staging, *CDKN3* gene expression and HPV type were included in univariate and multivariable Cox proportional hazards regression models. The statistical significance between the mean differences in cell lines experiments was calculated with the t test. All tests were 2 sided, and p values less than 0.05 were considered statistically significant. Data analysis was performed using Sigma Stat and SPSS ver. 17 software.

## Results

### *CDKN3* gene expression in cervical cancer

**Fig 1:**
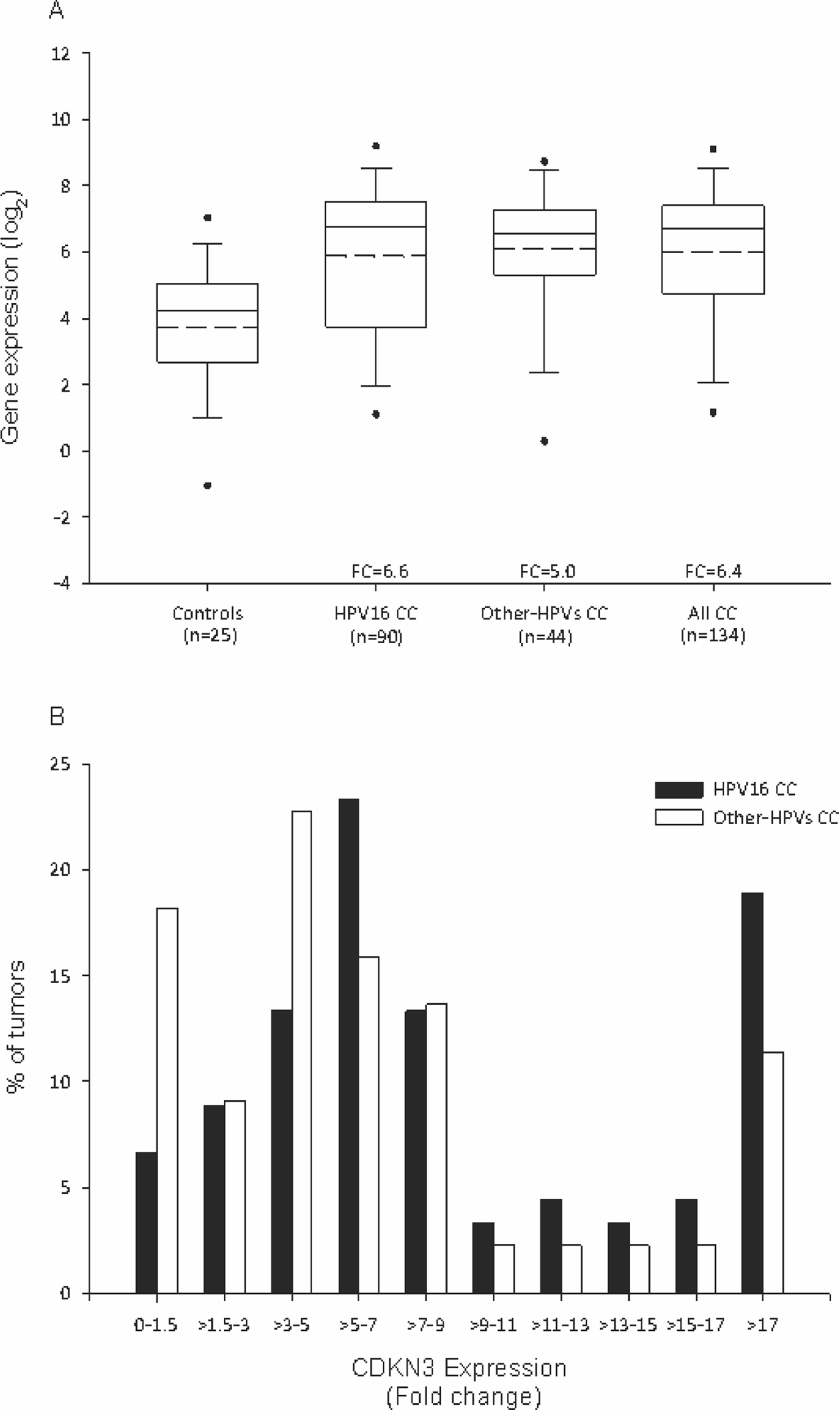
*CDKN3* gene expression in normal cervical epithelium and cervical cancer samples. The expression of cyclin-dependent kinase inhibitor 3 (*CDKN3*) was measured by RT-qPCR in 25 normal healthy cervix samples and 134 cervical cancer (CC) samples positive for human papillomavirus (HPV)16 (n = 90) or for other HPV types (n = 44), including 18, 31, 33, 35, 45, 51, 52, 53, 58, 59, and 68. (A) Intensity of gene expression, expressed in Log2 values, in box plots. The upper and lower boundaries of the boxes represent the 75th and 25th percentiles, respectively. The black and dotted lines within the boxes represent the median and mean values, respectively, and the whiskers represent the minimum and maximum values that lie within 1.5x the interquartile range from the end of the box. Values outside this range are represented by black circles. The fold change (FC) was calculated by dividing the median of each CC group by the median of the control group. Statistical differences between groups were calculated using the nonparametric Mann-Whitney U test. (B) Frequency (%) distribution of *CDKN3* FCs, which were calculated in each tumor considering the median of the control group.

Box plots (Fig 1A) clearly show that *CDKN3* expression was significantly higher in CC than in healthy cervical tissue (FC = 6.4, p = 8 x 10^−6^, MW test). Moreover, when cancer specimens were grouped according to HPV16 status, a difference in expression, compared to the control group, was maintained in both CC positive for HPV16 (FC = 6.6; p = 8 x 10^−5^, MW) and CC positive for other HPV types (FC = 5; p = 8 x 10^−6^, MW) (Fig 1A). Although *CDKN3* expression was found to be downregulated (FC < 1) or the FC value was equal to or less than 1.5 in 10.4% (n = 14) of tumors, mainly from the tumors positive for HPV types other than HPV16 (p<0.05, Chi-square test; Fig 1B), these data support the conclusion that *CDKN3* expression was increased in most CC independent of the HPV type.

### Association of *CDKN3* gene expression, clinical stage and viral type with survival

We investigated whether upregulation of *CDKN3* was associated with decreased survival time in 121 CC patients, and if any effects on survival were dependent of clinical stage and HPV type. To establish a separation line between dead and live patients and the potential value of *CDKN3* expression as a marker for survival, an average cut-off FC was calculated using the ROC curves (see Material and Methods). The average cut-off obtained was FC = 17. Of the 121 samples, 83 were positive for HPV 16 and 38 for other HPV types (S2 Table). The median time of follow up was 64 months from the initial diagnosis. Clinical stages (FIGO) of the 121 patients were IA1 (n = 1), IB1 (n = 35), IB2 (n = 29), IIA (n = 7), IIB (n = 35), IIIB (n = 9), and IV (n = 5). The overall survival of all patients was 71.9% and for FIGO clinical stages IA1/IB1, IB2, IIA, IIB, IIIB, and IV were 94.4%, 79.3%, 57.1%, 57.1%, 55.6%, and 20%, respectively. These differences were statistically significant (p = 1.2 x 10^−6^, log-rank test, S2A Fig).

**Fig 2:**
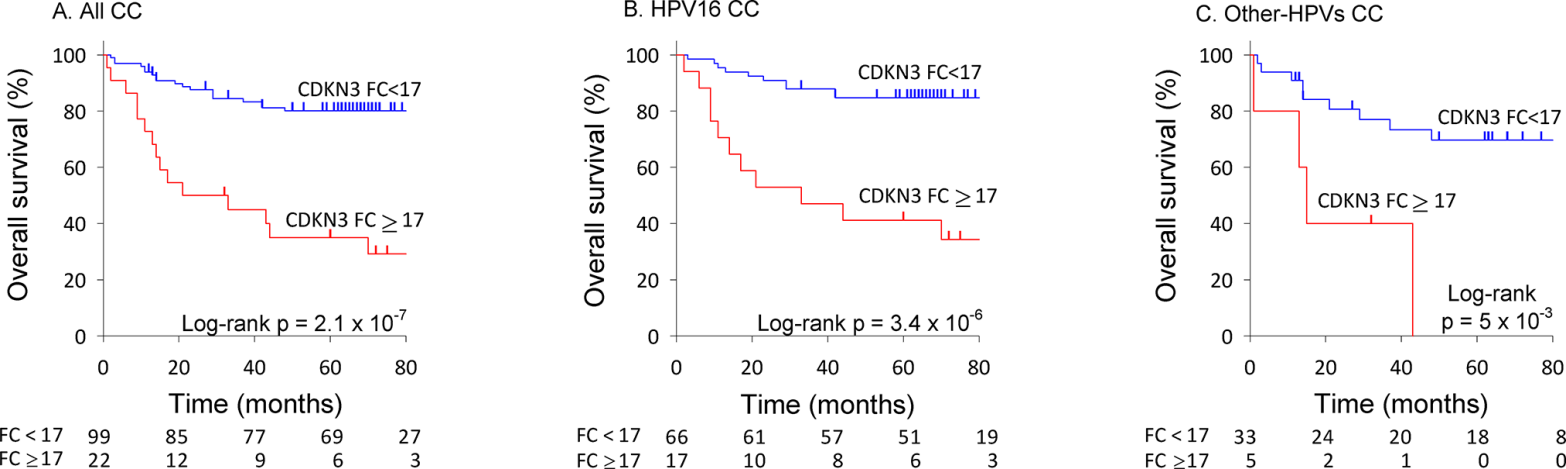
Survival analysis of women with cervical cancer according to *CDKN3* expression. The Kaplan-Meier curves for cyclin-dependent kinase inhibitor 3 (CDKN3) expression are shown. Patients were followed up for at least 60 months. Overall survival analyzed with Kaplan-Meier curves is shown for CDKN3 expression status, (A) all CC sample set, (B) HPV16-positive CC cases, (C) CC positive for HPVs other than HPV16. In all panels, the p-value was calculated by comparing the curves with the log-rank test. The number of patients at risk in each time intervals are noted in the tables below the curves. Censored patients are labeled with vertical bars (see material and methods).

The patients with high expression of *CDKN3* gene (FC ≥ 17) had a lower survival rate than those who had lower expression of *CDKN3* (p = 2.1 x 10^−7^, log-rank test; Fig 2A). The overall survival rate of patients with high expression levels of *CDKN3* was only 31.8% and the median survival time of the patients who died (68.2%) was 14 months. In contrast, the overall survival rate of patients with low expression levels of *CDKN3* (FC < 17) was 80.8% and the median survival time of women who died (19.2%) was 19 months.

The survival analysis by *CDKN3* expression level was performed in patients divided into two groups according to HPV16 status. For either groups of patients the overall survival was much lower in patients with high expression of *CDKN3* (p < 1 x 10^−2^, log-rank test; Fig 2B and 2C). On the other hand, there was no difference in the survival rate between patients positive for HPV16 and other HPV types (S2B Fig), neither between patients infected with HPV types from specie 9 (HPV16-related group) and patients infected with viruses from other species (S2C Fig).

The *CDKN3* expression FC, FIGO stage, and HPV type were analyzed individually and together in a Cox proportional hazards regression model. Due to the small sample size of some FIGO stages, cases were regrouped into 3 groups. Group I (n = 65) included clinical stages IA1, IB1 and IB2, group II (n = 42) included stages IIA and IIB, and group III (n = 14) included stages IIIB and IV. The hazard ratio (HR) for FC ≥ 17 of *CDKN3* expression was 5.0 (95% CI: 2.5–10, p = 3.3 x 10^−6^; Table 1). Taking the group I as a reference, the HR was 4.1 for group II (95% CI: 1.8–9.3, p = 1.0 x 10^−3^) and 6.9 for group III (95% CI: 2.6–18.4, p = 1.2 x 10^−4^). Taking the group positive for HPV16 as the reference, the HR for other HPV-positive CC was 0.6 (95% CI: 0.3–1.3, p = 1.9 x 10^−1^; Table 1), confirming no difference between the two groups of tumors. It is noteworthy that the regrouped FIGO stages were differentially distributed between patients with high and low *CDKN3* (p < 0.05, Pearson chi-squared test). While the frequency distribution of regrouped stages in high *CDKN3* tumors followed a normal curve with stage II at the top (50%), that in low *CDKN3* tumors decreased from stage I (59.6%) to stage III (9.1%). Therefore, to determine whether the influence of *CDKN3* expression in survival was independent of FIGO stage, both variables were included in the same Cox model. The HR for *CDKN3* remained significant (HR = 3.3, 95% CI: 1.6–6.8, p = 9.2 x 10^−4^; Table 1). These data indicate that the shorter survival of patients with high levels of *CDKN3* expression was independent of the FIGO clinical stage and HPV type.

**Table 1:** Univariate and multivariate survival analysis of patients with CC including *CDKN3* expression, clinical stage and HPV type.

Variable	*n*	Univariate (HR)^a^	p^b^	Confidence Interval 95%	Multivariate (HR)^c^	p^b^	Confidence Interval 95%
*CDKN3* expression (FC)^d^							
Low (< 17)	99	1			1		
High (≥ 17)	22	5	3.3 x 10^−6^	2.5–10.0	3.3	9.2 x 10^−4^	1.6–6.8
FIGO							
IB1/IB2	65	1			1		
IIA/IIB	42	4.1	1.0 x 10^−3^	1.8–9.3	3.1	9.6 x 10^−3^	1.3–7.3
IIIB/IVB	14	6.9	1.2 x 10^−4^	2.6–18.4	4.5	3.9 x 10^−3^	1.6–12.7
Viral type							
HPV16	83	1					
Other HPVs^e^	38	0.6	1.9 x 10^−1^	0.3–1.3			

a. Unadjusted Hazard Ratio. Univariate analysis was performed for each variable.

b. Cox proportional hazards model.

c. Adjusted Hazard Ratio. Multivariate analysis was performed considering CDKN3 expression (FC) and FIGO.

d. FC, fold change, expression of *CDKN3* in tumors in relation to the mean in the control group.

e. Positive tumors for HPVs 18, 31, 33, 35, 45, 51, 52, 53, 58, 59 and 68.

### Effect of inhibition of *CDKN3* gene expression on proliferation, migration and invasion of cell lines derived from CC

**Fig 3:**
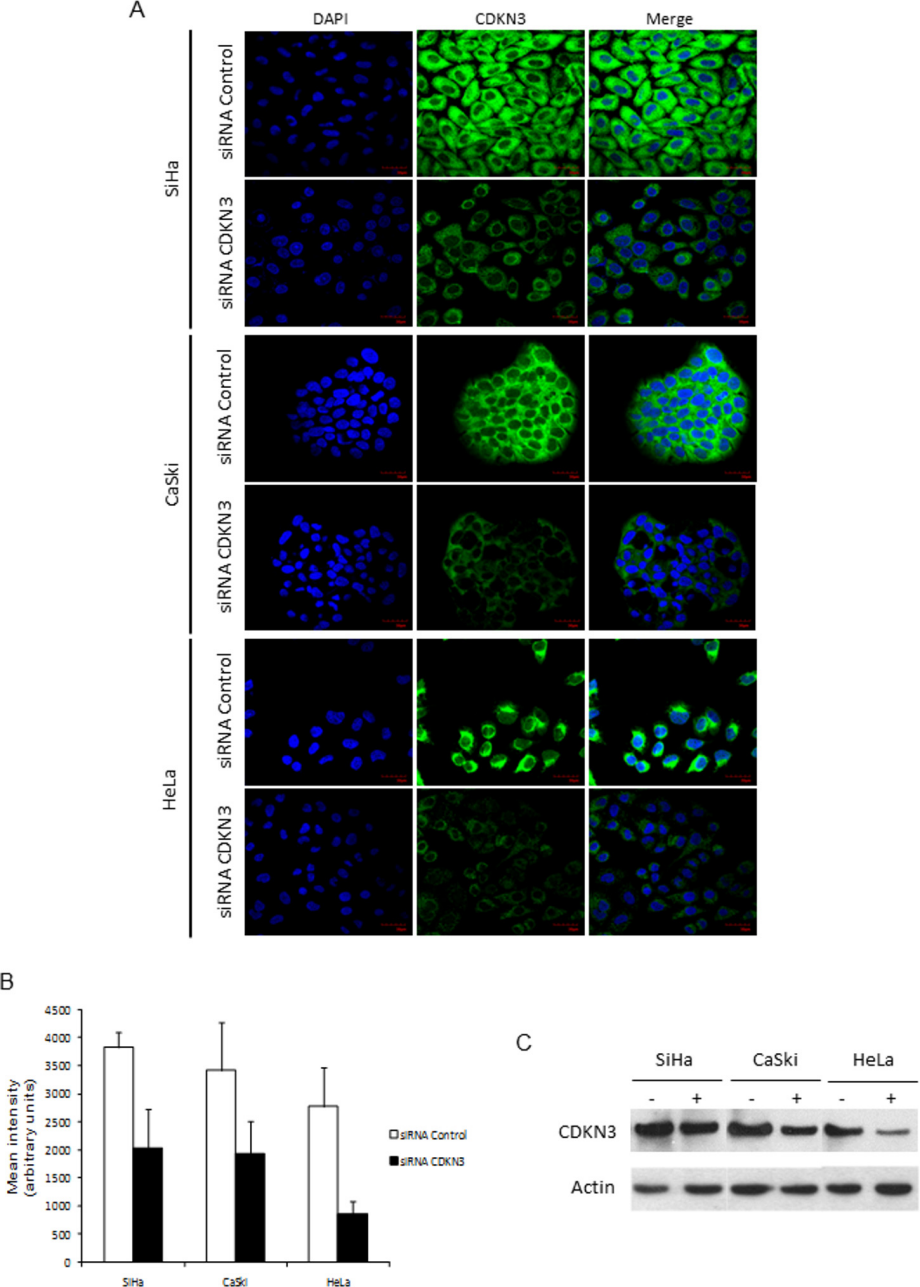
Detection of CDKN3 protein by immunofluorescence and Western blot in cell lines derived from CC transfected with specific siRNAs against *CDKN3* or scrambled siRNAs. Cell lines derived from cervical cancer (CC) positive for human papilloma virus (HPV) 16 (CaSki, SiHa) and HPV18 (HeLa) were transfected with specific cyclin-dependent kinase inhibitor 3 *(CDKN3)* or scrambled siRNAs. Cells were harvested at 96 h after transfection and stained for CDKN3 protein. (A) Immunofluorescence staining for CDKN3 protein in SiHa, CaSki and HeLa cell lines using an anti-CDKN3 primary antibody and FITC-conjugated secondary antibody. Images were photographed at 60× magnification using a fluorescence microscope Olympus FV 1000. (B) Quantification of fluorescence intensity of anti-CDKN3 antibody-stained cells. The green fluorescence intensity was quantified using ImageJ software. Values represent the mean ± S.D. of 140 fields measured in each experiment. The statistical significance between the differences was calculated using the t test. (C) Expression of CDKN3 protein examined using western blot in SiHa, CaSki, and HeLa cell lines transfected with random siRNAs (-) and with specific *CDKN3* siRNAs (+) with actin as internal control.

To investigate whether this gene is essential for proliferation, migration, and invasion of cancer cells, we inhibited *CDKN3* gene expression in cell lines derived from CC (HeLa, CaSki, SiHa) using specific siRNAs. The optimal concentrations of siRNAs that maximally decreased *CDKN3* expression were 80 nM for SiHa and 100 nM for CaSki, and HeLa cell lines. With these concentrations, there was an average reduction of 47.7% in *CDKN3* mRNA (p < 1 x 10^−4^, t test; Table 2) and 53% in CDKN3 protein (p < 1 x 10^−10^, t test; Fig 3A and 3B) expression in the three cell lines.

**Table 2:** *CDKN3* gene expression decreased in cervical cancer cell lines transfected with specific siRNAs against *CDKN3*.

	mRNA level^a^			
	Mean ± S.D.			
Cell line	siRNA control^b^	siRNA *CDKN3* ^c^	% of decrease^d^	p value^e^
CaSki	21.6 ± 2.1	8.6 ± 2.4	60.2	3 x 10^−5^
HeLa	211.3 ± 17.1	109.1 ± 15.4	48.3	8 x 10^−7^
SiHa	264.9 ± 31.5	173.1 ± 17.3	34.7	8 x 10^−7^

a. Level of mRNA normalized with an internal control (*GAPDH*).

b. Cells transfected with a mix of 3 scrambled siRNAs. The optimal concentration of siRNAs that greatest decreases the *CDKN3* messenger was 80 nM for SiHa and CaSki, and 100 nM for HeLa cell lines.

c. Cells transfected with a mix of 3 specific siRNAs against *CDKN3*. The optimal concentration of siRNAs that greatest decreases the CDKN3 messenger was 80 nM for SiHa and CaSki, and 100nM for HeLa cell lines.

d. % of Decrease = 100—(siRNA *CDKN3*/siRNA Control)*100

e. t test.

**Fig 4:**
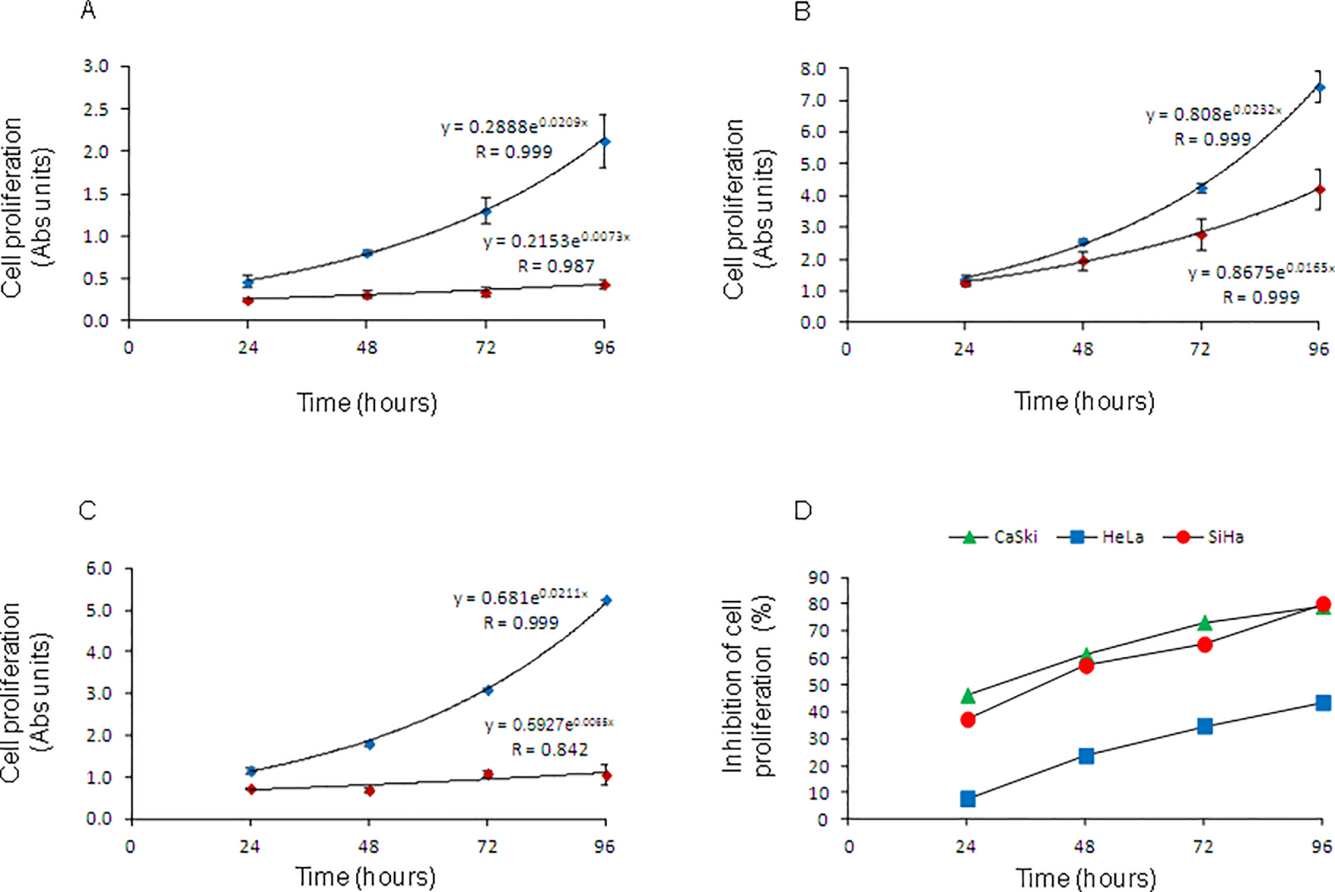
Cell proliferation curves of CC-derived cell lines transfected with specific siRNAs against *CDKN3* or scrambled siRNAs. Cell lines were transfected with specific cyclin-dependent kinase inhibitor 3 *(CDKN3)* (red diamonds) or scrambled siRNAs (blue diamonds). Cells were harvested at 24, 48, 72, and 96 h after transfection. Cell proliferation was measured using the MTT method, and the plates were read with a spectrophotometer at 560 nm. The experiments were repeated two times by triplicate, and the figure shows the data of one experiment. Mean ± standard deviation of triplicates, growth trend line, and growth model equations are shown for CaSki (A), HeLa (B) and SiHa (C). Cell growth follows an exponential trend predicted by the equation y_t_ = y_0 *_ e^rx^, where y_t_ = absorbance at specific time, x = incubation time in hours, y_0_ = a constant absorbance at time = 0, and r = rate growth. In panel D, we calculated the percentage of cell-proliferation inhibition of CaSki, HeLa and SiHa cell lines at 24, 48, 72, and 96 h post-transfection. At each time point, the mean absorbance of triplicates of cells transfected with the scrambled siRNAs was set as 100%.

Cell lines transfected with scrambled siRNAs showed typical exponential growth, indicating that the transfection with the control siRNAs did not affect cell growth (blue diamonds in Fig 4A–4C). In contrast, the cell proliferation decreased significantly in the three cell lines after they were transfected with specific siRNAs against *CDKN3* (red diamonds in Fig 4A–4C). Cell growth inhibition was observed from the first time point (24 h) in all but one cell line (HeLa), and increased linearly with the incubation time (Fig 4D). The highest level of inhibition was reached at 96 h, the last time point measured. The highest percent inhibition was observed in SiHa (80%), then in CaSki (79%) and finally in HeLa (43%) cells (Fig 4D). Consequently, cell doubling time increased in the three cell lines, most notably in SiHa, in which the doubling time increased three-fold. The doubling time of non-transfected SiHa cells or those transfected with irrelevant siRNAs was 32.9 h, whereas the doubling time of cells transfected with *CDKN3* specific siRNAs increased up to 106.6 h (Table 3).

**Table 3:** Doubling time increased in cervical cancer cell lines transfected with specific siRNAs against *CDKN3*.

	Doubling time^a^ (hours)		
Cell line	siRNA control^b^	siRNA *CDKN3* ^c^	Duplication rate^d^
CaSki	33.2	95.0	2.6
HeLa	29.9	42.0	1.4
SiHa	32.9	106.6	3.2

a. Calculated using the equations of exponential growth, included in Fig 4.

b. Cells transfected with a mix of 3 scrambled siRNAs

c. Cells transfected with a mix of 3 specific siRNAs against *CDKN3*.

d. Duplication rate = siRNA *CDKN3*/siRNA Control.

**Fig 5:**
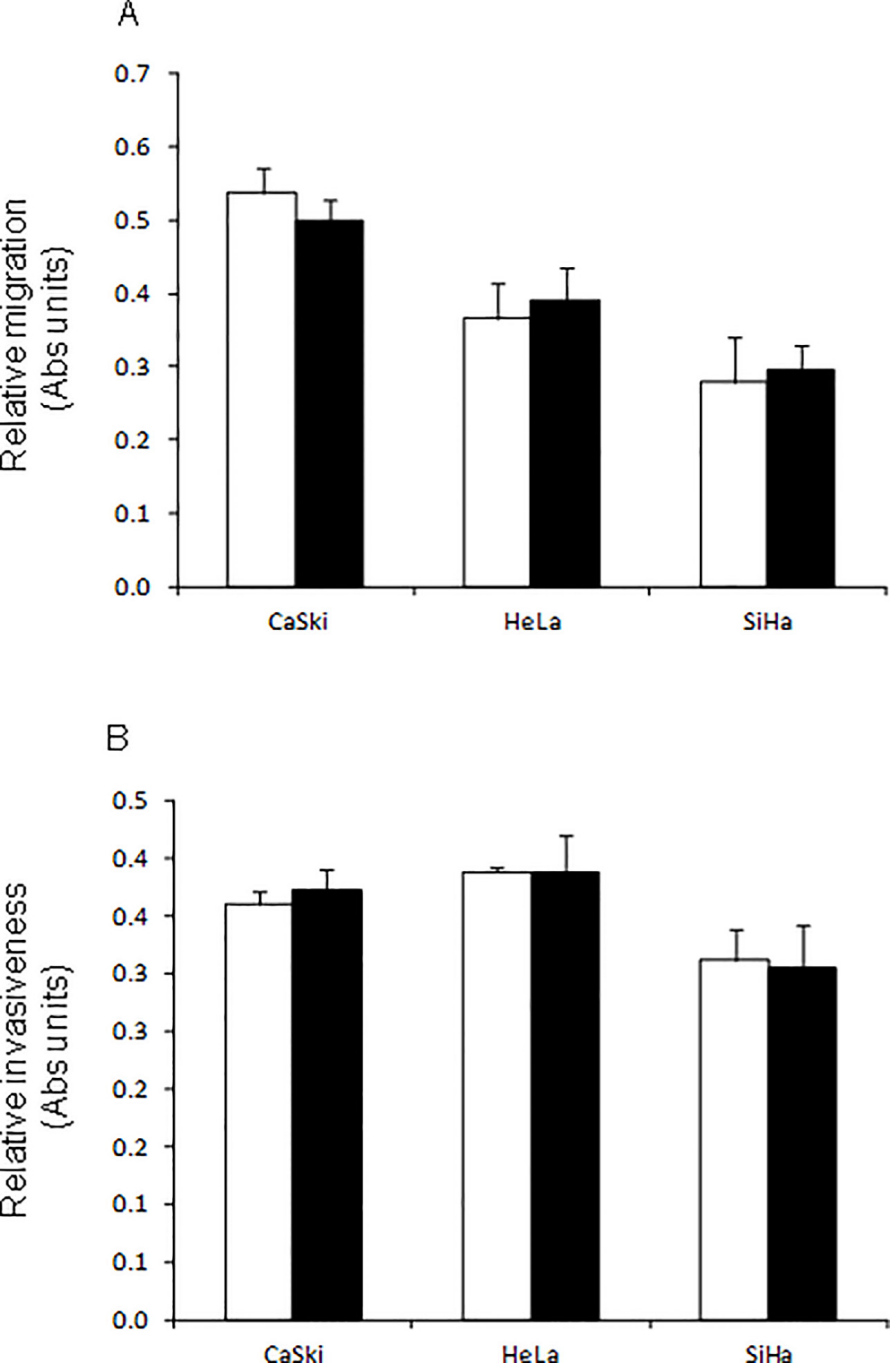
Cell migration and invasion of cervical cancer (CC)-derived cell lines transfected with specific siRNAs against cyclin-dependent kinase inhibitor 3 *(CDKN3)* or scrambled siRNAs. Cell lines were transfected with specific *CDKN3* (black bars) or scrambled siRNAs (white bars). Cells were harvested 48 h after transfection. Cell migration (A) and invasion (B) was assayed in a 24-well transwell polycarbonate membrane inserts and in a 24-well transwell matrigel invasion chamber, respectively. After incubation time, cells that migrated through the filters or invaded through the matrigel membranes were collected and stained with MTT colorimetric assay. The experiments were repeated two times by triplicate, and the figure shows the mean ± S.D. of one experiment.

The abilities of cell lines transfected with *CDKN3* siRNAs to migrate through a porous membrane (PET; 8.0 micron pores) and invade through a Matrigel membrane were similar to those of cells transfected with control siRNAs (Fig 5). This indicates that the decrease of *CDKN3* gene expression does not affect the migration and invasion capacity of these cell lines.

### Analysis of mRNA and protein variants of *CDKN3* gene

**Fig 6:**
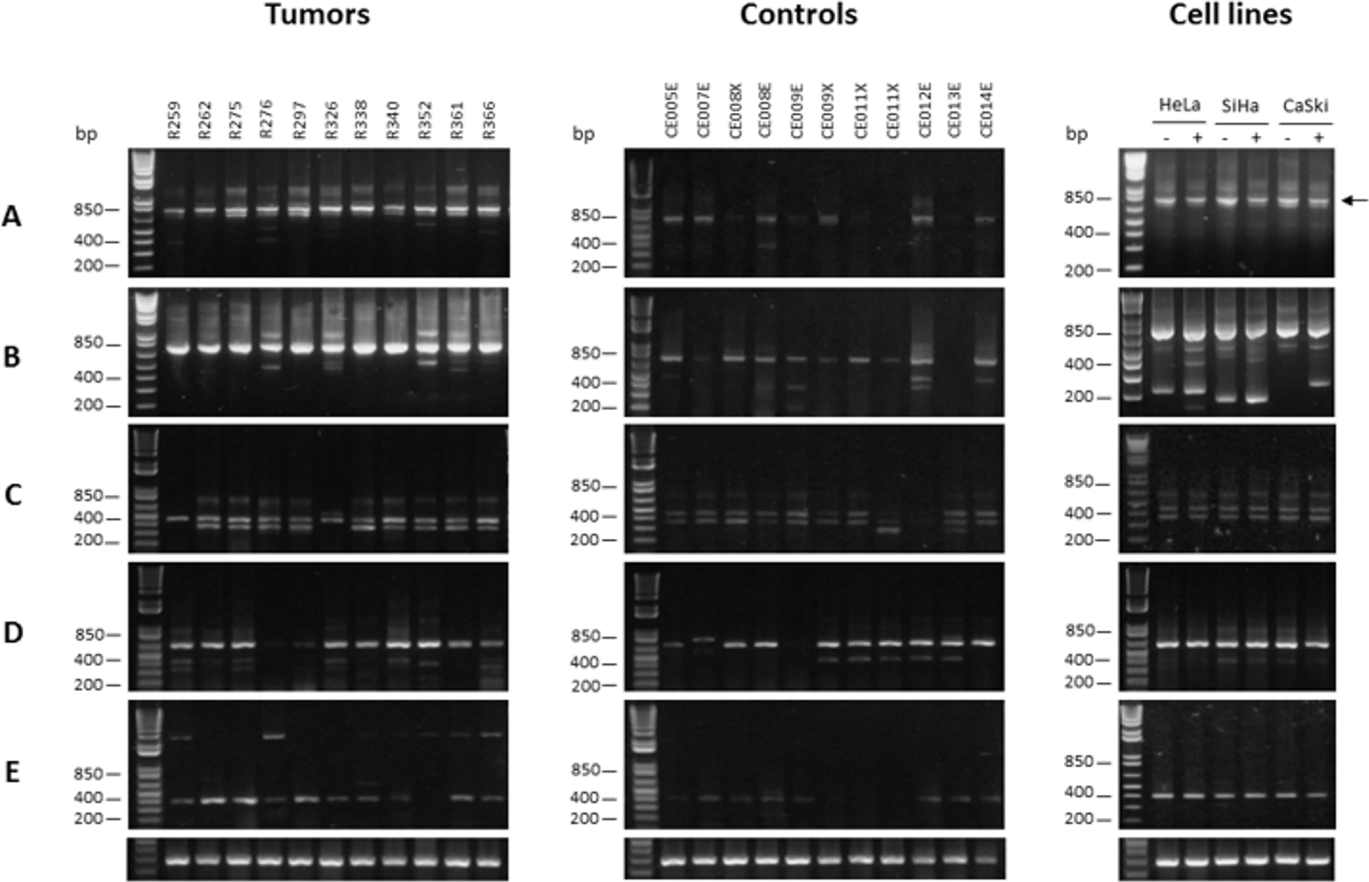
Analysis of *CDKN3* transcripts. Figure shows gel electrophoresis of mRNA variants of *CDKN3* gene identified in CC, controls, and cell lines using reverse transcription-polymerase chain reaction (RT-PCR). Panel A and B show wild-type (wt) *CDKN3* mRNA transcript. (A) RT-PCR was performed with external primers F1/R1 (756 bp, black arrow). (B) A 2-μL sample of 1:5 dilution of reactions of (A) were re-amplified using the nested primers F2/R5. In some samples, other weak transcripts were observed below wt*CDKN3* transcript (721 bp) including variants cx1, cx2, cx3, cx4, and cx5 (200, < 200, 400, 450, and 500 bp, respectively). (C) RT-PCR identified the “f” variant (453 bp) using primers F1/R9f. Most samples showed an additional lower band (cx6 variant, 370 bp). (D) RT-PCR identified “i” variant (633 bp) using primers F6i/R5. (E) RT-PCR identified “k” variant (392 bp) using primers F4/R5. Lower panel shows RT-PCR amplification of glyceraldehyde 3-phosphate dehydrogenase (*GAPDH*) gene as internal control. Cell lines were transfected with random siRNAs (-) or specific CDKN3 siRNAs (+), treated, and then incubated as described in Material and methods. RT-PCR was performed on RNAs extracted 48 h after transfections.

**Fig 7:**
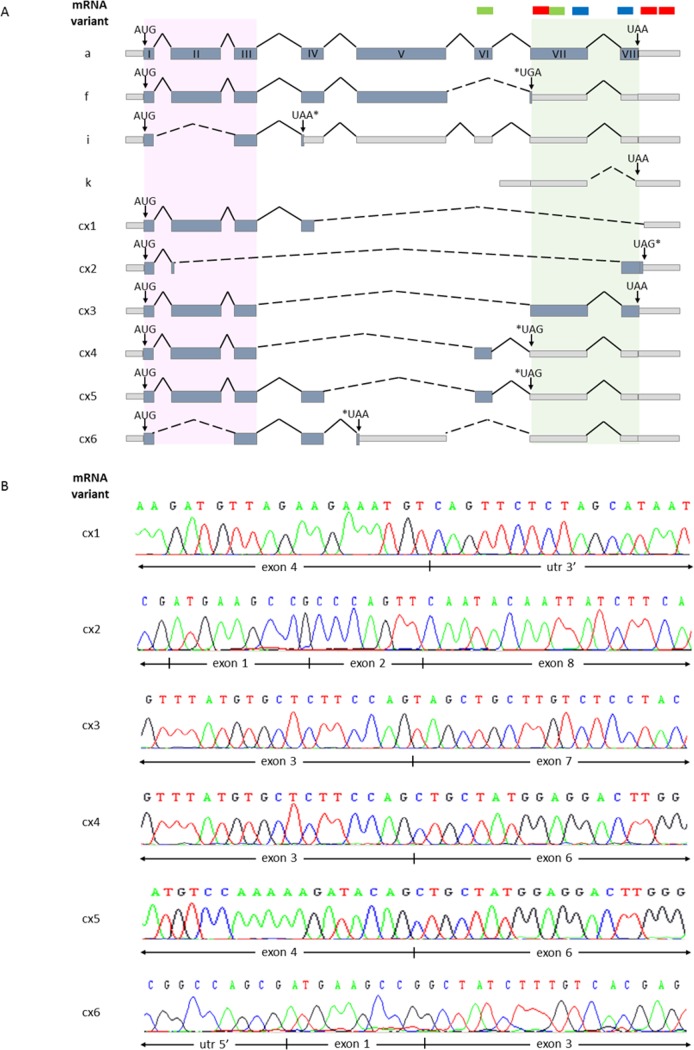
Sequencing analysis of *CDKN3* transcripts. All *CDKN3* transcripts found in analyzed samples were sequenced. (A) Graphical representation of sequencing findings in transcripts detected in samples including exons, introns, alternative splicings, and stop codons. Variant “a” represents wild-type (wt) CDKN3 mRNA and variants f, i, and k are transcripts previously reported that were also detected in our samples. Graphics of newly detected transcripts not previously reported are also shown (cx1, cx2, cx3, cx4, cx5, and cx6) based on sequences, which are partially shown in (B). Gray light boxes represent the 3ʹUTR, 5ʹUTR, and nontranslated sequences. Coding exons are represented by light blue boxes and introns are depicted by continuous black lines. Alternative splicings are represented by black dashed lines. Start codons (AUG) are shown at the beginning of every transcript. Normal stop codons (UAA) and stop codons generated by alternative splicing (*) are shown. Pink shaded rectangle in the 5ʹʹ region depicts the coding region of first 34 amino acids necessarily for CDK2 interaction. Shaded green rectangle in the 3ʹ region represents carboxyl terminal region of CDKN3, which participates in stabilization of CDK2 interaction. Green, blue, and red bars above transcripts in (A) indicate recognition sites of TaqMan probes, anti-CDKN3 antibody, and siRNA-CDKN3, respectively.

The *CDKN3* mRNA variants were analyzed in 45 tumors, 22 controls, and the three cell lines with RT-PCR. The transcript variants were identified by electrophoresis or sequencing of the amplified cDNA bands, or both. The wild-type *CDKN3* (wt*CDKN3*) transcript was found in all tumors and cell lines as well as most controls (86.3%) analyzed (S3 Table). The wt*CDKN3* corresponded to the superior 756 bp band (transcript “a”) in panel A of Fig 6. A second band was detected just below the wt*CDKN3* but did not correspond to any *CDKN3* transcript according to the cDNA sequence (data not shown). Below these bands, tenuous bands were observed in some samples and cell lines (panel A of Fig 6). To amplify these bands, a nested PCR was performed using internal primers (F2 and R5 in S1 Table, Panel B). Panel B shows the wt*CDKN3* transcript (721 bp) as the main band, but in many samples including the cell lines, there were several lower sized bands. Five of these bands (< 200, 200, 400, 450, and 500 bp) were sequenced (Fig 7) and named cx2, cx1, cx3, cx4, and cx5, respectively in Fig 7. Three of them excluded the VII and VIII exons from an open reading frame (ORF); one by splicing (cx1) and two by presenting early stop codons (cx4 and cx5, Fig 7A). The transcript cx2 excluded exon VII by splicing and only transcript cx3 presented exons VII and VIII in an ORF. These two exons along with N-terminal portion of CDKN3 are involved in interactions with CDK2 [30]. Although there were differences in the frequencies of these five transcripts between CC tumors and controls and between live and deceased patients, these differences were not statistically significant (see S3 Table).

From the transcripts previously reported ([28], S3 Table), besides the wt*CDKN3* transcript, only the “f" (panel C in Fig 6), “i” (panel D in Fig 6), and “k” (panel E in Fig 6) transcripts were found either in tumors, controls, or in cell lines. Together with the “f” transcript (453 bp), a second unreported band of 370 bp (named cx6 in Fig 7) with similar intensity to the “f” band, was detected in most samples and cell lines. This band showed an early stop codon excluding the V, VI, VII, and VIII exons from an ORF (Fig 7). The frequency of all transcripts was similar for tumors and controls and no statistically significant differences were found. However, the signals of all but one (“i”) of the *CDKN3* transcripts, mainly wtCDKN3, were weaker in the controls than they were in tumors (Fig 6 panels A-C, and E). The intensity of the “i” transcript was similar for tumors and controls (Fig 6 panel D). The primers and probe of the TaqMan assay used for the qPCR were located in exon VI and VII and these exons are present only in the main transcripts “a” and “i”. Therefore, the difference in *CDKN3* expression between controls and tumors measured using qPCR represented mainly the variations in the “a” transcript. The *CDKN3* transcripts were investigated in the cell lines transfected with unspecific (lines labeled as “-” in Fig 6) or specific *CDKN3* siRNAs (lines labeled as “+” in Fig 6). In these experiments, only the wt*CDKN3* (labeled with an arrow in Fig 6A) clearly decreased after the transfection with specific *CDKN3* siRNAs compared with the unspecific siRNAs. CDKN3 protein was analyzed using western blot in the three cell lines transfected with specific and unspecific *CDKN3* siRNAs. We only detected the wt protein band in these experiments (34 kDa, in Fig 3C). Similar to the wt*CDKN3* transcript, the wtCDKN3 protein decreases after transfection with specific siRNAs (Fig 3C). These data are in agreement with the results observed in the immunofluorescence analysis (Fig 3A).

### Cellular localization of CDKN3 protein in cell lines and CC, and control tissues using immunofluorescence and immunohistochemistry

**Fig 8:**
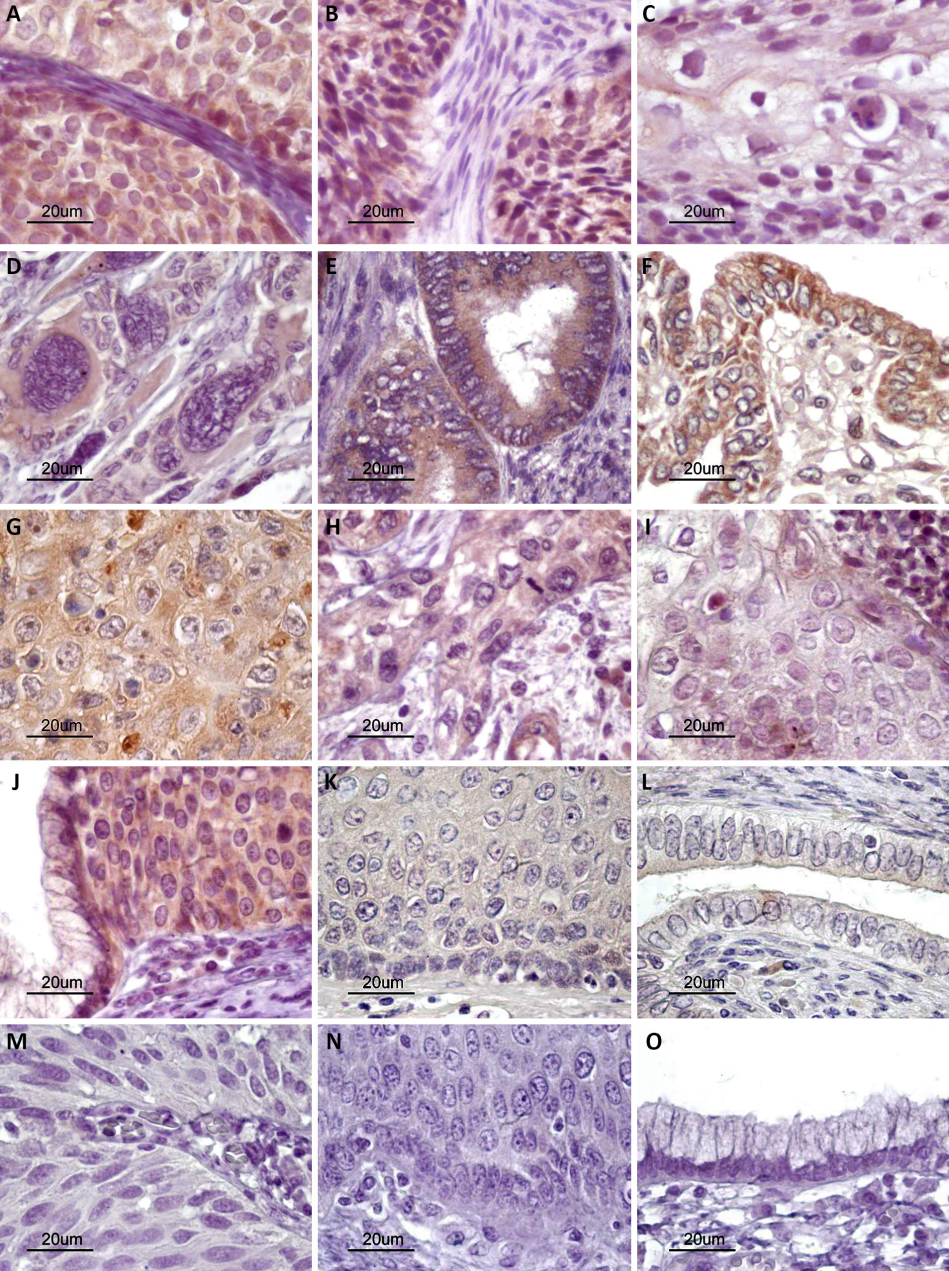
Histological analysis of CDKN3 protein expression. CDKN3 protein expression was determined by immunohistochemistry using formalin-fixed paraffin-embedded tissue sections. Representative experiments in HPV16-positive squamous cell carcinomas (A–D) and adenocarcinomas (E and F), and squamous cell carcinomas positive for other HPVs (G–I) are shown. Specific signals are shown as brown staining (counterstained with hematoxylin, original magnification ×800; bars, 20 μm). High-grade squamous intraepithelial lesion (J), normal cervical epithelium (K and L), and experimental controls without primary antibody (M–O) are also shown.

It is quite interesting that the CDKN3 protein was found localized exclusively in the cytoplasm of cells in the three cell lines explored using immunofluorescence (Fig 3A). To investigate the cellular localization of the protein in invasive tumors and normal cervix, CDKN3 was determined in 37 invasive tumors and 21 controls using immunohistochemistry. The percentage of tumors positive for CDKN3 was 89.2%. Although most controls showed CDKN3 signals (80%), the signals were much weaker than they were in the tumors (≤ 1 +). This difference in positivity was consistent with the data obtained with the qRT-PCR. Signals were observed in both the cytoplasm and nucleus. However, in HPV16-positive tumors (n = 22) the signals were stronger (3+ vs. 1+) and were predominantly in the cytoplasm rather than in the nucleus (64.5 ± 39 vs. 33.2 ± 37%, p < 0.05, t test, Fig 8, panels A–F). In tumors positive for HPVs other than HPV16 (n = 15), the signals were weaker than they were in HPV16-positive tumors (1+ vs. 3+); however, the distribution was similar in the cytoplasm and nucleus (66.7 ± 42 and 74 ± 27%, Fig 8, panels G–I).

## Discussion

In this study, we showed that about 70% of CC patients over expressing *CDKN3* gene died within two years of diagnosis, independent of the clinical stage and HPV type. We also found that inactivation or partial inhibition of *CDKN3* gene expression dramatically decreases cancer cell proliferation *in vitro*. Based on the study results, we can conclude that the *CDKN3* mRNA expression level might be a good predictive marker for evaluating patient survival and tumor aggressiveness. We can also conclude that the *CDKN3* gene, at the mRNA level, could be a good therapeutic target for tumor growth inhibition. Several other previously identified markers have been associated with poor CC patient survival: LIM kinase 1 [31], galectin 7 [32], miRNA-224 [33], NF-kB [34], and Caspase-3 [35]. However, none of these has been associated with an overall death rate higher than 45% compared to 70% for CDKN3 in this study. Although these differences could depend on the populations studied, the distance between these markers and CDKN3 was very obvious.

The association of *CDKN3* with decreased survival of CC patients and cancer cell proliferation clearly indicate that this gene might be involved in CC progression. Although we cannot disregard the role of *CDKN3* in tumor migration and invasion, the partial inhibition of CDKN3 expression (35–50%) suggests its involvement in tumor progression may be by inducing cell proliferation through a direct action on the cell cycle. Other signals, not related to *CDKN3*, may be central to the regulation of cell migration and invasion in CC [36, 37]. *CDKN3* has been found to be upregulated in breast, prostate, ovary, lung, renal, glioblastoma, liver, neuroendocrine, and oral cancers [27, 38–46]. In agreement with our data, upregulation of *CDKN3* gene, together with other genes, has been associated with decreased survival of patients with lung cancer [40] and astrocytoma [43]. Furthermore, *CDKN3* inhibition in many cell lines decreases cell proliferation, suggesting that this gene acts as an oncogene [47]. However, overexpression of *CDKN3* has been associated with the inhibition of cell proliferation in glioblastoma [42, 48] and leukemic [49] cell lines. In addition, *CDKN3* has been proposed to be a tumor suppressor gene of brain tumors [43, 48] and of mitosis control [48]. In fact, *CDKN3* protein binds to Cdk2 and dephosphorylates Thr160 when the associated cyclin subunit is degraded. Because the phosphorylation of Thr160 is necessary for full Cdk2 activity and cell cycle progression, dephosphorylation of Thr160 inhibits cell cycle progression [48]. However, in some tumors, some spliced or aberrant CDKN3 proteins were able to compete with the wt CDKN3 protein for the Cdk2-binding site, thereby avoiding the dephosphorylation of Thr160 [30, 43]. In the present study, we did not find mutated or aberrant CDKN3 transcripts, and in all CC samples and cell lines analyzed, the wt*CDKN3* transcript was predominant; however, some samples showed other known spliced mRNA variants (f, i, and k). New mRNA splicing variants, with early stop codons were also detected in some samples and cell lines. However, they were scarce and were detected only after a nested PCR was performed. In fact, in the western blot analysis we only detected the wt protein in cell lines. Although we could not explored the CDKN3 protein in tumor samples using western blotting, the cell line data and predominance of the wt*CDKN3* mRNA variant in tumors suggests that it was the wt CDKN3 protein that dominated in the CC samples and cell lines. Therefore, the inhibition mechanism mediated by competition between aberrant or spliced mRNA variants appeared to be absent in CC. On the other hand, the high-risk HPV oncoproteins stimulate the cells to exit the G1 phase and enter the cell cycle by promoting the inhibition or degradation of RB, p53, and p27 proteins. However, there is no evidence that these viral proteins also inhibit CDKN3 protein.

In this study, we showed conclusively that the CDKN3 protein was located exclusively in the cytoplasm of the three CC cell lines explored. In the CC tumors, it was also observed mainly in the cytoplasm, although the protein signals could be seen in the nucleus as well. The depletion of CDKN3 from the nucleus or its relocation to the cytoplasm appeared to be a compensatory mechanism to avoid the dephosphorylation of CDK2 by CDKN3. In fact, preliminary results showed no changes in the proportion of phosphorylated and unphosphorylated CDK2 protein at Thr^160^ in cell lines transfected with irrelevant or specific *CDKN3* siRNAs (data not shown). It is not known whether this relocation of CDKN3 to the cytoplasm is related to the viral proteins or is a regulatory mechanism of cancer cells that confers a growth selective advantage.

Interestingly, the wt*CDKN3* transcript was the one that diminished after the CC cell lines were transfected with the *CDKN3* specific siRNAs, as shown in Fig 6. These results suggest that the decrease in the wt transcript and its corresponding protein, located mainly in cytoplasm, is likely related to the inhibition or decrease of cell proliferation *in vitro* after the inhibition of the *CDKN3* gene. Therefore, other functions not related to *CDK2* inhibition could be involved. In fact, CDKN3 protein, a dual-specificity protein phosphatase of the Cdc14 phosphatase group, also appears to be essential in the regulation of mitosis exit. It interacts with CDK1 (CDC2) and inhibits its activity, and dephosphorylates many of the proteins phosphorylated by CDK1 [47, 48]. CDKN3 and other Cdc14 phosphatases appear to be essential for antagonizing Cdk activity in late mitosis, allowing cells to exit mitosis and enter cytokinesis. The regulation of cytokinesis may be the one conserved function of the Cdc14 phosphatases [48]. Therefore, the inhibition of the *CDKN3* gene would prevent or impair the exit from the M phase, to start a new cell cycle. However, it is still necessary to demonstrate the phase of the cell cycle that is altered when *CDKN3* is downregulated, mainly using fluorescence-activated cell sorting (FACS). In models in which *CDKN3* functions as a tumor suppressor gene, as in the leukemic cell line K562, the over expression of wt*CDKN3* delays the G1/S transition but had no significant effect on the timing of the G2/M/G1 progression [49]. Clarifying the role of *CDKN3* in cervical cancer is important to design appropriate strategies to target *CDKN3*.

The potential use of the *CDKN3* expression profile to select patients who need to be treated with more aggressive treatment is very promising. For patients with high levels of *CDKN3* expression (FC ≥ 17), there are at least three additional treatment options. First, the inclusion of neoadjuvant chemotherapy with paclitaxel and carboplatin [50, 51], second, the use of angiogenesis blockers, such as bevacizumab [52, 53], and third, the use of epidermal growth factor receptor inhibitors like erlotinib [54]. However, appropriate clinical trials should investigate the clinical value of measuring *CDKN3* mRNA as an indicator for additional neoadjuvant chemotherapy or specific target cancer therapy.

Several factors make *CDKN3* a potential target for CC treatment. First, *CDKN3* is over expressed in CC, on average 6-fold more than in the normal cervix. Second, *CDKN3* is involved in mitosis. Third, inhibition of *CDKN3* gene expression reduced cell proliferation by an average of 67% in cell lines derived from CC. It is important to note that despite reducing by about half the *CDKN3* mRNA and protein expression with specific siRNAs, the reduction of proliferation was on average 67%. This suggests that a greater inhibition of *CDKN3* would increase the reduction in proliferation. Although inhibition of *CDKN3* seems to have no effect on migration and invasion of the cell lines derived from CC, the effect in decreasing cell proliferation alone makes *CDKN3* a potential therapeutic target. In fact, if we could block 100% cell proliferation, we would secondarily block migration and invasion, as there would be no cells to migrate and invade. Furthermore, there are no specific targeted anticancer drugs that block all biological processes related to cancer, and yet, they are used to treat patients [55, 56]. However, we do not know whether the effect of *CDKN3* on the inhibition of cell proliferation *in vitro*, would be reflected in reduction of tumor growth in a mouse model. Moreover, we do not know the effect of inhibiting the *CDKN3* gene on proliferation of normal cells. However, because *CDKN3* is over-expressed in cancer cells, it is expected that its effect in normal cells would be minimal.

The use of specific siRNAs to block or inhibit the activity of a gene is a well-established methodology for *in vitro* models, particularly to investigate gene function [57–59]. However, the use of siRNAs as therapeutic agents in tumor models has not been entirely successful, especially for invasive or metastatic cancers [60, 61]. The first stage to design or identify selective or specific anti-cancer drugs is to identify targets present in cancer tissues and absent in normal tissues. Clearly, *CDKN3* seems to be a target in CC, but further study is needed to identify or develop either a small molecule drug that inhibits CDKN3 function specifically in neoplastic cells or an effective systemic delivery strategy to introduce *CDKN3*-specific siRNAs into cancer cells [62].

Although at the selected cut-off (FC = 17) the specificity of *CDKN3* was very high (92%; the % of surviving patients who had FC < 17), the sensitivity of *CDKN3* was low (44.1%; the % of dead patients who had FC ≥ 17). Therefore, additional markers are needed to increase the sensitivity of *CDKN3*. In addition to the reports published by our group [21, 63], there is only one other study [64] in which *CDKN3* has been found to be overexpressed in CC. It is important to emphasize that more studies are needed to completely understand the role of *CDKN3* in cervical cancer and determine if it is associated with low survival of patients with CC in other populations as well.

## Conclusions

The results presented here will help to develop new strategies for the treatment of CC. In this study, we showed that about 70% of CC patients over expressing *CDKN3* gene died within two years of diagnosis, independent of the clinical stage and HPV type. We also found that inactivation or partial inhibition of *CDKN3* gene expression dramatically decreases cancer cell proliferation *in vitro*. The potential use of the *CDKN3* expression profile to select patients who need to be treated with more aggressive treatment is very promising. On the other hand, as *CDKN3* seems to be indispensable for cancer cell proliferation, it could be a good therapeutic target for tumor growth inhibition.

## Supporting Information

S1 Fig Specificity of the secondary antibody. Cell lines derived from cervical cancer (CC) positive for human papilloma virus (HPV) 16 (CaSki, SiHa) and HPV18 (HeLa) were transfected with specific cyclin-dependent kinase inhibitor 3 (CDKN3) or scrambled siRNAs. Cells were harvested at 96 h after transfection and stained as in Fig 3, but without the primary antibody against CDKN3. Images were photographed at 60× magnification using an Olympus FV 1000 fluorescence microscope.
(TIF)

S2 Fig Survival analysis of women with cervical cancer according to FIGO staging, HPV type, and HPV specie. The Kaplan-Meier curves for cervical cancer (CC) staging, human papilloma virus (HPV) type, and HPV specie are shown. Patients were followed up for at least 60 months. Overall survival analyzed with Kaplan-Meier curves is shown for CC patients classified by stage (A), HPV type (B) or HPV specie (C). In all panels, the p-value was calculated by comparing the curves with the log-rank test. The number of patients at risk in each time intervals are noted in the tables below the curves. Censored patients are labeled with vertical bars (see material and methods).
(TIF)

S1 Table List of primers used for identification of CDKN3 mRNA variants. (DOCX)

S2 Table Clinical data and fold change of *CDKN3* expression of 134 cervical cancer patients. A, A total of 134 samples were screened for the expression of CDKN3 by RT-qPCR, but only 121 patients were included in the survival study. Patients that did not receive treatment (n = 8) or lost during the follow-up in the first 10 months (n = 5) were not included in the survival analysis (marked with an asterisk). B, ACC, Adeno-cell carcinoma. SCC, Squamous-cell carcinoma. ASCC, Adenosquamous-cell carcinoma. UND, Undifferentiated. C, HT, radical hysterectomy. Tele, teletherapy. Brachy, brachytherapy. Chemo, chemotherapy with Cisplatin. D, Status alive was registered at the last follow up, death was caused by primary tumor of cervical cancer, except the case labeled with a double asterisk (R221), and unknown cases were lost during the follow up study. The cause of death of case labeled with a double asterisk was unknown. E, Fold change (FC) was calculated with the median values as follows: expression of CDKN3 in each tumor/expression of CDKN3 in the control set.
(DOCX)

S3 Table Frequency of different mRNA variants of *CDKN3* gene. A, The variants a, b, c, e, f, i, and k have been previously reported in other cancer types, whereas the variants cx1–cx6 are reported here for the first time. Variants cx1–cx5 were amplified in nested RT-PCRs from a 1:5 dilution of the reaction for variant a. Variant cx6 was amplified simultaneously and with the same primers used for variant f. B, The cell lines explored were CaSki, HeLa, and SiHa. C, The variant "a" is the wild type (wt) transcript of the CDKN3 gene.
(DOCX)

## Abbreviations

CC: Cervical cancer
HPV: Human papilloma virus
CDKN3: Cyclin-dependent kinase inhibitor 3
FIGO: Fédération Internationale de Gynécologie Obstétrique
GAPDH: Glyceraldehyde 3-phosphate dehydrogenase
RT-qPCR: Retro transcription-quantitative PCR
FC: Fold change
ROC: Receiver Operator Characteristic
MW: Mann–Whitney test
HR: Hazard ratio

## References

[1] Bruni L, Barrionuevo-Rosas L, Albero G, Aldea M, Serrano B, Valencia S, et al. ICO Information Centre on HPV and Cancer (HPV Information Centre). Human Papillomavirus and Related Diseases in the World. Summary Report 2014-02-20. [Data Accessed]

[2] Hwang SJ, Shroyer KR. Biomarkers of cervical dysplasia and carcinoma. J Oncol 2012;507286 10.1155/2012/507286 22131995 PMC3205687

[3] Ferlay J, Soerjomataram I, Ervik M, Dikshit R, Eser S, Mathers C, et al. GLOBOCAN 2012 v1.0, Cancer Incidence and Mortality Worldwide: IARC CancerBase No. 11 [Internet]. Lyon, France: International Agency for Research on Cancer; 2013. Available: http://globocan.iarc.fr, accessed on day/month/year.

[4] Crosbie E, Einstein M, Franceschi S, Kitchener HC. Human papillomavirus and cervical cancer. Lancet 2013;382(9895):889–99. 10.1016/S0140-6736(13)60022-7 23618600

[5] Schiffman M, Wentzensen N, Wacholder S, Kinney W, Gage JC, Castle PE. Human papillomavirus testing in the prevention of cervical cancer. J Natl Cancer Inst 2011;103:368–83. 10.1093/jnci/djq562 21282563 PMC3046952

[6] Kjaer SK, Sigurdsson K, Iversen OE, Hernandez-Avila M, Wheeler CM, Wheeler CM, et al A pooled analysis of continued prophylactic efficacy of quadrivalent human papillomavirus (Types 6/11/16/18) vaccine against high-grade cervical and external genital lesions. Cancer Prev Res (Phila) 2009;2(10):868–78. doi: 10.1158/1940-6207 2:868–78 19789295 10.1158/1940-6207.CAPR-09-0031

[7] Lehtinen M, Paavonen J, Wheeler CM, Jaisamrarn U, Garland SM, Castellsaqué X, et al Overall efficacy of VPH-16/18 AS04-adjuvanted vaccine against grade 3 or greater cervical intraepithelial neoplasia: 4-year end-of-study analysis of the randomized, double-blind PATRICIA trial. Lancet Oncol 2012;13(1):89–99. 10.1016/S1470-2045(11)70286-8 22075171

[8] Romanowski B. Long term protection against cervical infection with the human papillomavirus: review of currently available vaccines. Hum Vaccine 2011;7:161–9. 21307652 10.4161/hv.7.2.13690

[9] Cuzick J. Long-term cervical cancer prevention strategies across the globe. Gynecol Oncol 2010;117(2 Suppl):S11–4. 10.1016/j.ygyno.2010.01.025 20129652

[10] Markowitz LE, Dunne EF, Saraiya M, Chesson HW, Curtis CR, Unger ER. Quadrivalent Human Papillomavirus Vaccine: Recommendations of the Advisory Committee on Immunization Practices (ACIP). MMWR Recomm Rep 2007;56(RR-2):1–24. 17380109

[11] Natunen K, Lehtinen J, Namujju P, Sellors J, Lehtinen M. Aspects of prophylactic vaccination against cervical cancer and other human papillomavirus-related cancers in developing countries. Infect Dis Obstet Gynecol 2011;2011:675858 10.1155/2011/675858 21785556 PMC3140204

[12] Van de Velde N, Boily MC, Drolet M, Franco EL, Mayrand MH, Kliewer EV, et al Population-level impact of the bivalent, quadrivalent, and nonavalent human papillomavirus vaccines: a model-based analysis. J Natl Cancer Inst 2012;104(22):1712–23. 10.1093/jnci/djs395 23104323

[13] Guardado-Estrada M, Juárez-Torres E, Román-Bassaure E, Medina-Martinez I, Alfaro A, Benuto RE, et al The Distribution of High-Risk Human Papillomaviruses Is Different in Young and Old Patients with Cervical Cancer. PLoS ONE 2012;9(10):e109406. 10.1371/journal.pone.0109406 PMC4190176 25295590

[14] Andrae B, Andersson TM, Lambert PC, Kemetli L, Silfverdal L, Strander B, et al Screening and cervical cancer cure: population based cohort study. BMJ 2012;344:e900 10.1136/bmj.e900 22381677 PMC3291751

[15] Torti D, Trusolino L. Oncogene addiction as a foundational rationale for targeted anti-cancer therapy: promises and perils. EMBO Mol Med 2011;3(11):623–36. 10.1002/emmm.201100176 21953712 PMC3377106

[16] Knight Z, Lin H, Shokat KM. Targeting the cancer kinome through polypharmacology. Nature Cancer Rev 2010;10(2):130–7. 10.1038/nrc2787 20094047 PMC2880454

[17] Saxena R, Dwivedi A. ErbB family receptor inhibitors as therapeutic agents in breast cancer: current status and future clinical perspective. Med Res Rev 2012;32(1):166–215. 10.1002/med.20209 22183797

[18] Yarden Y, Pines G. The ERBB network: at last, cancer therapy meets systems biology. Nat Cancer Rev 2012;12(8):553–63. 10.1038/nrc3309 22785351

[19] Bryant C, Scriven K, Massey AJ. Inhibition of the checkpoint kinase Chk1 induces DNA damage and cell death in human Leukemia and Lymphoma cells. Molecular Cancer 2014;13:147 10.1186/1476-4598-13-147 24913641 PMC4082411

[20] Yu Y, Wang XY, Sun L, Wang YL, Wan YF, Li XQ, et al Inhibition of KIF22 suppresses cancer cell proliferation by delaying mitotic exit through upregulating CDC25C expression. Carcinogenesis 2014;35(6):1416–25. 10.1093/carcin/bgu065 24626146

[21] Espinosa AM, Alfaro A, Roman-Besaure E, Guardado-Estrada M, Palma Í, Serralde C, et al Mitosis is a source of potential markers for screening and survival and therapeutic targets in cervical cancer. PLoS ONE 2013;8(2):e55975 10.1371/journal.pone.0055975 23405241 PMC3566100

[22] Pecorelli S. Revised FIGO staging for carcinoma of the vulva, cervix, and endometrium. Int J Gynaecol Obstet 2009;105(2):103–4. 19367689 10.1016/j.ijgo.2009.02.012

[23] Gravitt PE, Peyton CL, Alessi TQ, Wheeler CM, Coutlée F, Hildesheim A, et al Improved amplification of genital human papillomaviruses. J Clin Microbiol 2000;38: 357–61. 10618116 10.1128/jcm.38.1.357-361.2000 PMC88724

[24] Schmitt M, Dondog B, Waterboer T, Pawlita M. Homogeneous amplification of genital human alpha papillomaviruses by PCR using novel broad-spectrum GP5+ and GP6+ primers. J Clin Microbiol 2008;46(3):1050–9. 10.1128/JCM.02227-07 18199790 PMC2268381

[25] Yoshikawa H, Kawana T, Kitagawa K, Mizuno M, Yoshikura H, Iwamoto A. Detection and typing of multiple genital human papillomaviruses by DNA amplification with consensus primers. Cancer Sci 1991;82: 524–531. 1648051 10.1111/j.1349-7006.1991.tb01882.x PMC5918477

[26] Guardado-Estrada M, Medina-Martínez I, Juárez-Torres E, Roman-Bassaure E, Macías L, Alfaro A, et al The Amerindian mtDNA haplogroup B2 enhances the risk of HPV for cervical cancer: de-regulation of mitochondrial genes may be involved. J Hum Genet 2012;57(4):269–76. 10.1038/jhg.2012.17 22357541

[27] Yeh CT, Lu SC, Chen TC, Peng CY, Liaw YF. Aberrant transcripts of the cyclin-dependent kinase associated protein phosphatase in hepatocellular carcinoma. Cancer Res 2000;60(17):4697–4700. 10987270

[28] Thierry-Mieg D and Thierry-Mieg J. AceView: a comprehensive cDNA-supported gene and transcripts annotation, Genome Biology 2006;7 Suppl 1:S121–14. 16925834 10.1186/gb-2006-7-s1-s12 PMC1810549

[29] Mathew M, Zaineb K, Verma R. GM-CSF-DFF40: a novel humanized immunotoxin induces apoptosis in acute myeloid leukemia cells. Apoptosis 2013;18(7):882–95. 10.1007/s10495-013-0840-8 23529188

[30] Yeh CT, Lu SC, Chao CH, Chao ML. Abolishment of the interaction between cyclin-dependent kinase 2 and Cdk-associated protein phosphatase by a truncated KAP mutant. Biochem Biophys Res Commun 2003;305(2):311–4. 12745075 10.1016/s0006-291x(03)00757-5

[31] Chhavi, Saxena M, Singh S, Negi MP, Srivastava AK, Trivedi R, et al Expression profiling of G2/M phase regulatory proteins in normal, premalignant and malignant uterine cervix and their correlation with survival of patients. J Cancer Res Ther 2010;6(2):167–71. 10.4103/0973-1482.65242 20622363

[32] Tsai CJ, Sulman EP, Eifel PJ, Jhingran A, Allen PK, Daevers MT, et al Galectin-7 levels predict radiation response in squamous cell carcinoma of the cervix. Gynecol Oncol 2013;131(3):645–9. 10.1016/j.ygyno.2013.04.056 23643871

[33] Shen SN, Wang LF, Jia YF, Hao YQ, Zhang L, Wang H. Upregulation of microRNA-224 is associated with aggressive progression and poor prognosis in human cervical cancer. Diagn Pathol 2013;8:69 10.1186/1746-1596-8-69 23631806 PMC3661379

[34] Wu Z, Peng X, Li J, Zhang Y, Hu L. Constitutive Activation of Nuclear Factor κB Contributes to Cystic Fibrosis Transmembrane Conductance Regulator Expression and Promotes Human Cervical Cancer Progression and Poor Prognosis. Int J Gynecol Cancer 2013;23(5):906–15. 10.1097/IGC.0b013e318292da82 23640294

[35] Hu Q, Peng J, Liu W, He X, Cui L, Yang M, et al Elevated cleaved caspase-3 is associated with shortened overall survival in several cancer types. Int J Clin Exp Pathol 2014;7(8):5057–70. 25197379 PMC4152069

[36] Wan HY, Li QQ, Zhang Y, Tian W1, Li YN, Liu, et al MiR-124 represses vasculogenic mimicry and cell motility by targeting amotL1 in cervical cancer cells. Cancer Lett 2014;355(1):148–58. 10.1016/j.canlet.2014.09.005 25218344

[37] Feng M, Wang Y, Chen K, Bian Z, Jinfang Wu, Gao Q. IL-17A Promotes the Migration and Invasiveness of Cervical Cancer Cells by Coordinately Activating MMPs Expression via the p38/NF-κB Signal Pathway. PLoS 2014;9(9):e108502 10.1371/journal.pone.0108502 25250801 PMC4177222

[38] Lee SW, Reimer CL, Fang L, Iruela-Arispe ML, Aaronson SA. Overexpression of kinase-associated phosphatase (KAP) in breast and prostate cancer and inhibition of the transformed phenotype by antisense KAP expression. Mol Cell Biol 2000;20(5):1723–32. 10669749 10.1128/mcb.20.5.1723-1732.2000 PMC85355

[39] Li T, Xue H, Guo Y. CDKN3 is an independent prognostic factor and promotes ovarian carcinoma cell proliferation in ovarian cancer. Oncol Rep 2014;31(4):1825–31. 10.3892/or.2014.3045 24573179

[40] MacDermed DM, Khodarev NN, Pitroda SP, Edwards DC, Pelizzari CA, Huang L, et al MUC1-associated proliferation signature predicts outcomes in lung adenocarcinoma patients. BMC Med Genomics 2010;3:16 10.1186/1755-8794-3-16 20459602 PMC2876055

[41] Lai MW, Chen TC, Pang ST, Yeh CT. Overexpression of cyclin-dependent kinase associated protein phosphatase enhances cell proliferation in renal cancer cells. Urol Oncol 2012;30(6):871–8. 10.1016/j.urolonc.2010.09.010 21396835

[42] Lee J, Sung CO, Lee EJ, Do IG, Kim HC, SH, Yoon SH, et al Metastasis of neuroendocrine tumors are characterized by increased cell proliferation and reduced expression of the ATM gene. PLoS ONE 2012;7(4):e34456 10.1371/journal.pone.0034456 22485171 PMC3317775

[43] Yu Y, Jiang X, Schoch BS, Carroll RS, Black PM, Johnson MD. Aberrant Splicing of Cyclin-Dependent Kinase–Associated Protein Phosphatase KAP Increases Proliferation and Migration in Glioblastoma. Cancer Res 2007;67(1):130–8. 17210692 10.1158/0008-5472.CAN-06-2478

[44] Lin WR, Lai MW, Yeh CT. Cyclin-dependent kinase-associated protein phosphatase is overexpressed in alcohol-related hepatocellular carcinoma and influences xenograft tumor growth. Oncol Rep 2013;29(3):903–10. 10.3892/or.2012.2208 23292002 PMC3597585

[45] Xing C, Xie H, Zhou L, Zhou W, Zhang W, Ding S, et al Cyclin-dependent kinase inhibitor 3 is overexpressed in hepatocellular carcinoma and promotes tumor cell proliferation. Biochem Biophys Res Commun 2012;420(1):29–35. 10.1016/j.bbrc.2012.02.107 22390936

[46] Hunter KD, Thurlow JK, Fleming J, Drake PJ, Vass JK, Kalna G, et al Divergent routes to oral cancer. Cancer Res 2006;66(15):7405–7413. 16885335 10.1158/0008-5472.CAN-06-0186

[47] Berumen J, Espinosa AM, Medina I. Targeting CDKN3 in cervical cancer. Expert Opin Ther Targets 2014;18(10):1149–62. 10.1517/14728222.2014.941808 25152075

[48] Nalepa G, Barnholtz-Sloan J, Enzor R, Dey D, He Y, Gehlhausen JR, et al The tumor suppressor CDKN3 controls mitosis. J. Cell Biol 2013;201(7):997–1012. 10.1083/jcb.201205125 23775190 PMC3691455

[49] Chen Q, Chen K, Guo G, Li F, Chen C, Wang S, et al A critical role of CDKN3 in Bcr-Abl-mediated tumorigenesis. PLoS ONE 2014;9(10):e111611 10.1371/journal.pone.0111611 25360622 PMC4216094

[50] Singh R, Chander S, Mohanti BK, Pathy S, Kumar S, Bhatla N, et al Neoadjuvant chemotherapy with weekly paclitaxel and carboplatin followed by chemoradiation in locally advanced cervical carcinoma: A pilot study. Gynecol Oncol 2013;129(1):124–8. 10.1016/j.ygyno.2013.01.011 23353129

[51] McCormack M, Kadalayil L, Hackshaw A, Hall-Craggs MA, Symonds RP, Warwick V, et al A phase II study of weekly neoadjuvant chemotherapy followed by radical chemoradiation for locally advanced cervical cancer. Br J Cancer 2013;108(12):2464–9. 10.1038/bjc.2013.230 23695016 PMC3694233

[52] Suh D, Kim J-W, Kang S, Kim HJ, Lee KH. Major clinical research advances in gynecologic cancer in 2013. J Gynecol Oncol 2014;25(3):236–48. 10.3802/jgo.2014.25.3.236 25045437 PMC4102743

[53] Jackson M, Rusthoven Ch, Fisher Ch, Schefter TE. Clinical potential of bevacizumab in the treatment of metastatic and locally advanced cervical cancer: current evidence. OncoTargets Ther 2014;7:751–9. 10.2147/OTT.S49429 24876784 PMC4037327

[54] Nogueira-Rodrigues A, Moralez G, Grazziotin R, Carmo CC, Small IA, Alves FV, et al Phase 2 trial of erlotinib combined with cisplatin and radiotherapy in patients with locally advanced cervical cancer. Cancer 2014;120(8):1187–93. 10.1002/cncr.28471 24615735

[55] Lewis Phillips GD, Li G, Dugger DL, Crocker LM, Parsons KL, Mai E, et al Targeting HER2-positive breast cancer with trastuzumab-DM1, an antibody-cytotoxic drug conjugate. Cancer Res 2008;68(22):9280–90. 10.1158/0008-5472 19010901

[56] Weaver BA. How Taxol/paclitaxel kills cancer cells. Mol Biol Cell 2014;25(18):2677–81. doi: 10.1091/mbc 25213191 25213191 10.1091/mbc.E14-04-0916 PMC4161504

[57] Cao T, Gao Z, Gu L, Chen M, Yang B, Cao K, et al AdipoR1/APPL1 Potentiates the Protective Effects of Globular Adiponectin on Angiotensin II-Induced Cardiac Hypertrophy and Fibrosis in Neonatal Rat Atrial Myocytes and Fibroblasts. PLoS ONE 2014;9(8):e103793 10.1371/journal.pone.0103793 25099270 PMC4123880

[58] Schimmack S, Taylor A, Lawrence B, Alaimo D, Schmitz-Winnenthal H, Büchler MW, et al A mechanistic role for the chromatin modulator, NAP1L1, in pancreatic neuroendocrine neoplasm proliferation and metastases. Epigenetics Chromatin 2014;7:15 10.1186/1756-8935-7-15 25071868 PMC4112619

[59] Wen X, Zhu J, Dong L, Chen Y. The role of c2orf68 and PI3K/Akt/mTOR pathway in human colorectal cancer. Med Oncol 2014;31(8):92 10.1007/s12032-014-0092-7 25023051

[60] Liu L, Liu X, Xu Q, Wu P, Zuo X, Zhang J, et al Self-assembled nanoparticles based on the c(RGDfk) peptide for the delivery of siRNA targeting the VEGFR2 gene for tumor therapy. Int J Nanomedicine 2014;9:3509–26. 10.2147/IJN.S63717 25114522 PMC4122582

[61] Pappano W, Zhang Q, Tucker L, Tse C, Wang J. Genetic inhibition of the atypical kinase Wee1 selectively drives apoptosis of p53 inactive tumor cells. BMC Cancer 2014;14:430 10.1186/1471-2407-14-430 24927813 PMC4229861

[62] Gavrilov K, Saltzman WM. Therapeutic siRNA: principles, challenges, and strategies. Yale J Biol Med 2012;85(2):187–200. 22737048 PMC3375670

[63] Medina-Martínez I, Barrón V, Roman-Bassaure E, Juárez-Torres E, Guardado-Estrada M, Espinosa AM, et al Impact of Gene Dosage on Gene Expression, Biological Processes and Survival in Cervical Cancer: A Genome-Wide Follow-Up Study. PLoS ONE 2014;9(5):e97842 10.1371/journal.pone.0097842 24879114 PMC4039463

[64] Santin AD, Zhan F, Bignotti E, Siegel ER, Cané S, Bellone S, et al Gene expression profiles of primary HPV16-and HPV18-infected early stage cervical cancers and normal cervical epithelium: identification of novel candidate molecular markers for cervical cancer diagnosis and therapy. Virology 2005;331(2):269–91. 15629771 10.1016/j.virol.2004.09.045

