# Supplementary figures and images for: CDKN3 mRNA as a Biomarker for Survival and Therapeutic Target in Cervical Cancer
Source: PLoS One. 2015 Sep 15;10(9):e0137397. doi: 10.1371/journal.pone.0137397 (PMC4570808; doi:10.1371/journal.pone.0137397)

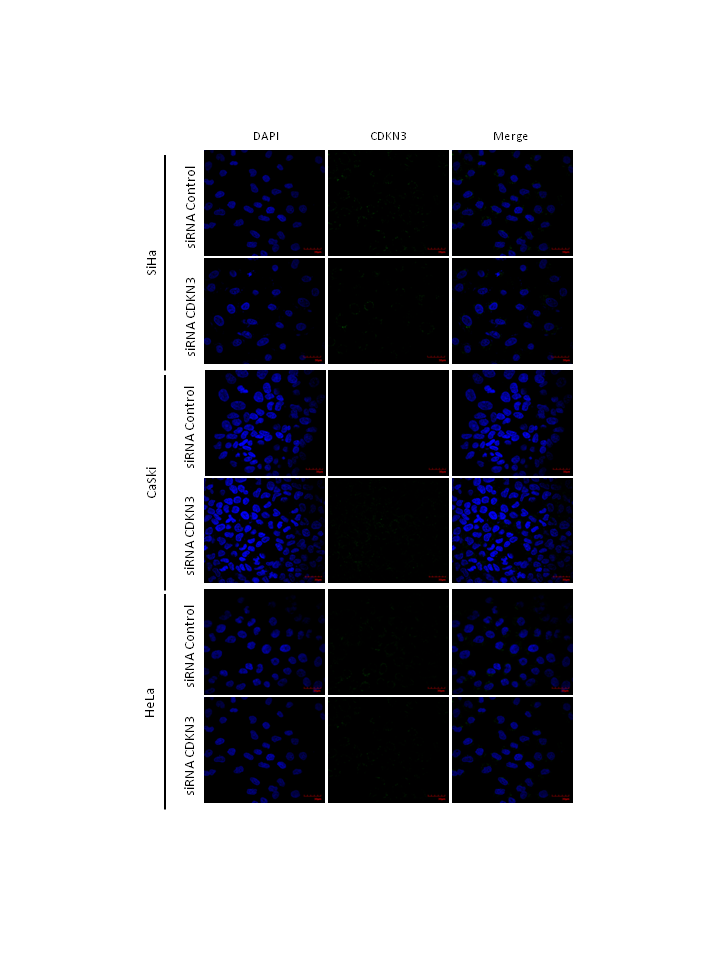

Supplement: S1 Fig — Cell lines derived from cervical cancer (CC) positive for human papilloma virus (HPV) 16 (CaSki, SiHa) and HPV18 (HeLa) were transfected with specific cyclin-dependent kinase inhibitor 3 (CDKN3) or scrambled siRNAs. Cells were harvested at 96 h after transfection and stained as in Fig 3, but without the primary antibody against CDKN3. Images were photographed at 60× magnification using an Olympus FV 1000 fluorescence microscope. (TIF) [file pone.0137397.s001.tif]

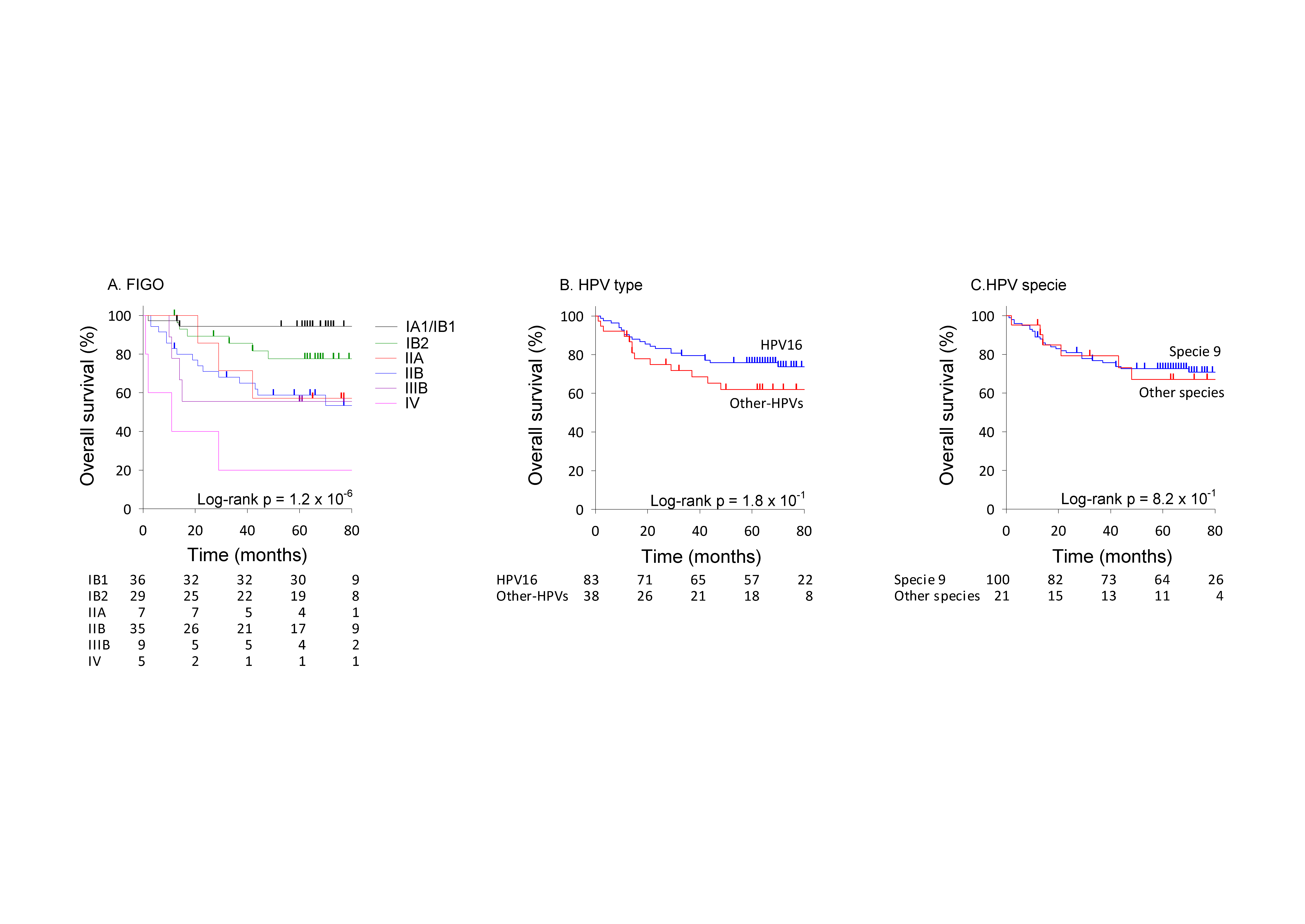

Supplement: S2 Fig — The Kaplan-Meier curves for cervical cancer (CC) staging, human papilloma virus (HPV) type, and HPV specie are shown. Patients were followed up for at least 60 months. Overall survival analyzed with Kaplan-Meier curves is shown for CC patients classified by stage (A), HPV type (B) or HPV specie (C). In all panels, the p-value was calculated by comparing the curves with the log-rank test. The number of patients at risk in each time intervals are noted in the tables below the curves. Censored patients are labeled with vertical bars (see material and methods). (TIF) [file pone.0137397.s002.TIF]
