# Supplementary material for: CDKN3 mRNA as a Biomarker for Survival and Therapeutic Target in Cervical Cancer
Source: PLoS One. 2015 Sep 15;10(9):e0137397. doi: 10.1371/journal.pone.0137397 (PMC4570808; doi:10.1371/journal.pone.0137397)
Supplement: S1 Table — (DOCX) [file pone.0137397.s003.docx]

**S1 Table. List of primers used by identification of CDKN3 mRNA variants.**

| Primer ID | Sequence 5’ – 3’ |
| --- | --- |
| F1* | ACT GGT CTC GAC GTG GGG CG |
| R1* | GTT GAT AAC ACT GGT GGT TTC |
| F2* | CCA GCG ATG AAG CCG CCC AG |
| R5 | TGG TTT CAT TTC AAT ACA AAT TAT GCT |
| R9f | GAC AAG CAG CTA TGT ATT AAG GTT T |
| F5-e | TGA AGC CGC CCA GTT CAA TAC AA |
| F6-i | GAT GAA GCC GGC TAT CTT TGT CA |
| F4 | GGA ACA ATT ACA CCA GTC TTC |

* Primers previously described.
