# Supplementary material for: CDKN3 mRNA as a Biomarker for Survival and Therapeutic Target in Cervical Cancer
Source: PLoS One. 2015 Sep 15;10(9):e0137397. doi: 10.1371/journal.pone.0137397 (PMC4570808; doi:10.1371/journal.pone.0137397)
Supplement: S2 Table — A, A total of 134 samples were screened for the expression of CDKN3 by RT-qPCR, but only 121 patients were included in the survival study. Patients that did not receive treatment (n = 8) or lost during the follow-up in the first 10 months (n = 5) were not included in the survival analysis (marked with an asterisk). B, ACC, Adeno-cell carcinoma. SCC, Squamous-cell carcinoma. ASCC, Adenosquamous-cell carcinoma. UND, Undifferentiated. C, HT, radical hysterectomy. Tele, teletherapy. Brachy, brachytherapy. Chemo, chemotherapy with Cisplatin. D, Status alive was registered at the last follow up, death was caused by primary tumor of cervical cancer, except the case labeled with a double asterisk (R221), and unknown cases were lost during the follow up study. The cause of death of case labeled with a double asterisk was unknown. E, Fold change (FC) was calculated with the median values as follows: expression of CDKN3 in each tumor/expression of CDKN3 in the control set. (DOCX) [file pone.0137397.s004.docx]

**S2 Table. Clinical data and fold change of *CDKN3* expression of 134 cervical cancer patients.**

| Sample^a^ | Histology^b^ | Tumor stage | Age (years) | Treatment^c^ | Follow up (months) | Status^d^ | HPV type | Fold Change^e^ |
| --- | --- | --- | --- | --- | --- | --- | --- | --- |
| R015 | SCC | IIB | 42 | TELE+HT | 66 | Alive | HPV16 | 11.9 |
| R035 | SCC | IB2 | 48 | TELE+CHEMO+HT | 73 | Alive | HPV16 | 3.4 |
| R052 | ACC | IIB | 54 | TELE+BRACHY+HT | 19 | Death | HPV16 | 6.8 |
| R057 | ACC | IB1 | 32 | HT | 93 | Alive | HPV16 | 8.7 |
| R070* | SCC | IIB | 74 | TELE+BRACHY+CHEMO | 4 | Unknown | HPV16 | 7.5 |
| R072 | SCC | IB1 | 61 | HT+TELE+BRACHY | 86 | Alive | HPV16 | 4.4 |
| R081 | ACC | IB1 | 41 | HT | 62 | Alive | HPV16 | 5.0 |
| R093 | ACC | IB1 | 57 | HT | 53 | Alive | HPV16 | 10.8 |
| R094 | SCC | IB1 | 45 | HT | 62 | Alive | HPV16 | 5.1 |
| R170 | SCC | IIB | 67 | TELE+CHEMO+HT | 90 | Alive | HPV16 | 5.0 |
| R183 | SCC | IB1 | 64 | TELE+BRACHY | 70 | Alive | HPV16 | 5.5 |
| R221 | SCC | IB2 | 41 | TELE+BRACHY+CHEMO | 33 | Death** | HPV16 | 5.6 |
| R232 | SCC | IB2 | 45 | TELE+BRACHY+CHEMO | 33 | Death | HPV16 | 22.0 |
| R240 | SCC | IIIB | 31 | TELE+BRACHY+CHEMO | 11 | Death | HPV16 | 30.5 |
| R245* | SCC | IB1 | 64 | UNTREATED | 19 | Death | HPV16 | 14.2 |
| R251 | SCC | IB1 | 61 | TELE+BRACHY | 14 | Unknown | HPV18 | 6.4 |
| R254* | SCC | IB1 | 23 | HT | 5 | Unknown | HPV45 | 4.1 |
| R255 | SCC | IIA | 45 | TELE+BRACHY+CHEMO | 42 | Death | HPV16 | 4.8 |
| R256 | SCC | IIA | 78 | TELE | 21 | Death | HPV51 | 7.4 |
| R257 | SCC | IB1 | 61 | TELE+BRACHY | 2 | Death | HPV16 | 41.4 |
| R258 | SCC | IB1 | 36 | HT+TELE+BRACHY | 68 | Alive | HPV16 | 5.5 |
| R259 | SCC | IVA | 46 | TELE+BRACHY+CHEMO | 91 | Alive | HPV16 | 325.3 |
| R260 | SCC | IB2 | 24 | TELE+BRACHY | 66 | Alive | HPV16 | 2.2 |
| R261 | SCC | IB1 | 43 | HT | 77 | Alive | HPV18 | 3.6 |
| R262 | SCC | IIB | 54 | TELE+BRACHY+CHEMO | 9 | Death | HPV16 | 602.0 |
| R264 | SCC | IIB | 49 | TELE+BRACHY+CHEMO | 64 | Alive | HPV16 | 16.9 |
| R265 | SCC | IB1 | 46 | TELE+BRACHY | 86 | Alive | HPV16 | 12.8 |
| R266 | SCC | IIA | 51 | TELE+BRACHY+CHEMO | 77 | Alive | HPV18 | 3.3 |
| R268 | SCC | IIB | 34 | TELE+BRACHY+CHEMO | 95 | Alive | HPV16 | 5.9 |
| R269* | SCC | IB1 | 66 | UNTREATED | 0 | Unknown | HPV16 | 2.0 |
| R271 | SCC | IIB | 56 | TELE+BRACHY+CHEMO | 83 | Alive | HPV31 | 1.5 |
| R275 | SCC | IIB | 54 | TELE+BRACHY+CHEMO | 70 | Death | HPV16 | 485.9 |
| R276 | SCC | IIB | 49 | HT+TELE+BRACHY+CHEMO | 21 | Death | HPV16 | 111.9 |
| R278 | SCC | IB1 | 54 | HT | 13 | Unknown | HPV45 | 4.4 |
| R282 | SCC | IIIB | 41 | TELE+BRACHY+CHEMO | 86 | Alive | HPV16 | 2.0 |
| R284 | ACC | IB2 | 33 | TELE+BRACHY+CHEMO | 75 | Alive | HPV16 | 17.6 |
| R289 | SCC | IIB | 44 | TELE+BRACHY+CHEMO | 12 | Unknown | HPV31 | 3.6 |
| R291* | SCC | IIB | 55 | UNTREATED | 33 | Death | HPV16 | 2.1 |
| R296 | SCC | IB2 | 48 | TELE+BRACHY+CHEMO | 69 | Alive | HPV16 | 6.4 |
| R297 | SCC | IIB | 47 | TELE+BRACHY+CHEMO | 94 | Alive | HPV16 | 302.4 |
| R307 | ACC | IIB | 74 | TELE+BRACHY | 50 | Alive | HPV58 | 13.7 |
| R308 | ACC | IB1 | 45 | TELE+BRACHY | 61 | Alive | HPV16 | 14.0 |
| Sample^a^ | Histology^b^ | Tumor stage | Age (years) | Treatment^c^ | Follow up (months) | Status^d^ | HPV type | Fold Change^e^ |
| R312 | ACC | IB2 | 34 | TELE+BRACHY+CHEMO | 68 | Alive | HPV16 | 8.7 |
| R315 | SCC | IIIB | 41 | TELE+BRACHY+CHEMO | 10 | Death | HPV16 | 6.3 |
| R316 | SCC | IB1 | 67 | TELE+BRACHY | 77 | Alive | HPV51 | 4.8 |
| R319 | SCC | IB1 | 47 | TELE+BRACHY | 89 | Alive | HPV68 | 8.3 |
| R322 | SCC | IIIB | 74 | TELE+BRACHY | 60 | Alive | HPV16 | 19.0 |
| R323* | SCC | IIIB | 78 | UNTREATED | 1 | Unknown | HPV33 | 5.4 |
| R324 | SCC | IB2 | 28 | TELE+BRACHY+CHEMO | 14 | Death | HPV16 | 30.5 |
| R326 | SCC | IIB | 66 | TELE+BRACHY+CHEMO | 37 | Death | HPV33 | 11.0 |
| R330 | SCC | IB1 | 72 | TELE+BRACHY | 73 | Alive | HPV16 | 16.4 |
| R333 | SCC | IIB | 56 | TELE+BRACHY+CHEMO | 81 | Alive | HPV16 | 5.7 |
| R335 | ACC | IB1 | 37 | HT+TELE+BRACHY+CHEMO | 65 | Alive | HPV16 | 10.5 |
| R336 | SCC | IB2 | 36 | TELE+BRACHY+CHEMO | 64 | Alive | HPV16 | 4.5 |
| R338 | ASCC | IB1 | 44 | TELE+BRACHY+CHEMO | 81 | Alive | HPV16 | 3.6 |
| R339 | SCC | IB2 | 31 | TELE+BRACHY+CHEMO | 13 | Death | HPV16 | 5.8 |
| R340 | SCC | IB1 | 29 | HT | 63 | Alive | HPV45 | 0.1 |
| R343 | SCC | IB1 | 49 | HT+TELE+BRACHY | 90 | Alive | HPV31 | 2.2 |
| R352 | SCC | IIB | 43 | TELE+BRACHY+CHEMO | 44 | Death | HPV16 | 37.0 |
| R354 | SCC | IIB | 56 | TELE+BRACHY | 89 | Alive | HPV16 | 2.5 |
| R359 | ACC | IB2 | 34 | TELE+BRACHY+CHEMO | 17 | Death | HPV16 | 18.4 |
| R361 | SCC | IB2 | 28 | TELE+BRACHY+CHEMO | 84 | Alive | HPV16 | 10.1 |
| R366 | SCC | IB2 | 41 | TELE+BRACHY+CHEMO | 82 | Alive | HPV16 | 3.1 |
| R367 | SCC | IB2 | 45 | TELE+BRACHY+CHEMO | 42 | Death | HPV16 | 5.7 |
| R368 | SCC | IIIB | 36 | TELE+BRACHY+CHEMO | 90 | Alive | HPV16 | 2.4 |
| R369 | SCC | IB1 | 50 | HT | 65 | Alive | HPV16 | 6.1 |
| R373 | SCC | IIB | 41 | TELE+BRACHY+CHEMO | 9 | Death | HPV16 | 96.6 |
| R374 | SCC | IIB | 60 | HT+TELE+BRACHY+CHEMO | 89 | Alive | HPV16 | 3.0 |
| R375 | ACC | IB1 | 54 | HT | 86 | Alive | HPV16 | 3.2 |
| R376 | SCC | IB2 | 46 | TELE+BRACHY+CHEMO | 88 | Alive | HPV16 | 1.3 |
| R378 | SCC | IB2 | 42 | TELE+BRACHY+CHEMO | 67 | Alive | HPV16 | 6.5 |
| R379 | SCC | IB2 | 59 | TELE+BRACHY | 12 | Unknown | HPV18 | 7.2 |
| R380* | SCC | IIIB | 64 | UNTREATED | 12 | Death | HPV16 | 6.4 |
| R381 | SCC | IB2 | 46 | TELE+BRACHY+CHEMO | 91 | Alive | HPV31 | 14.9 |
| R385 | SCC | IB2 | 47 | TELE+BRACHY | 48 | Death | HPV45 | 8.1 |
| R386 | SCC | IB1 | 73 | TELE+BRACHY | 70 | Alive | HPV16 | 6.5 |
| R390 | SCC | IB1 | 51 | TELE+BRACHY+CHEMO | 64 | Alive | HPV16 | 8.3 |
| R392 | SCC | IIIB | 69 | TELE+BRACHY | 61 | Alive | HPV16 | 1.5 |
| R393 | SCC | IB1 | 42 | TELE+BRACHY | 68 | Alive | HPV33 | 1.4 |
| R394 | ACC | IIB | 49 | TELE+BRACHY+CHEMO | 11 | Death | HPV33 | 0.1 |
| R396* | ACC | IB2 | 53 | TELE+BRACHY+CHEMO | 7 | Unknown | HPV16 | 4.2 |
| R397 | SCC | IB2 | 42 | TELE+BRACHY+CHEMO | 63 | Alive | HPV16 | 7.9 |
| R398 | ACC | IB2 | 46 | TELE+BRACHY+CHEMO | 64 | Alive | HPV45 | 5.8 |
| R399 | SCC | IIB | 42 | TELE+BRACHY+CHEMO | 58 | Alive | HPV16 | 5.5 |
| R400 | SCC | IB2 | 32 | TELE+BRACHY+CHEMO | 88 | Alive | HPV59 | 7.5 |
| R401 | SCC | IA1 | 73 | HT | 71 | Alive | HPV16 | 7.0 |
| Sample^a^ | Histology^b^ | Tumor stage | Age (years) | Treatment^c^ | Follow up (months) | Status^d^ | HPV type | Fold Change^e^ |
| R403 | SCC | IIB | 34 | TELE+BRACHY+CHEMO | 64 | Alive | HPV16 | 7.3 |
| R405 | SCC | IB1 | 51 | TELE+BRACHY+CHEMO | 85 | Alive | HPV33 | 4.0 |
| R407 | ACC | IIB | 53 | TELE+BRACHY | 43 | Death | HPV51 | 20.2 |
| R409 | SCC | IB2 | 68 | TELE+BRACHY+CHEMO | 42 | Alive | HPV16 | 11.1 |
| R411 | SCC | IB1 | 34 | HT+TELE+BRACHY | 84 | Alive | HPV16 | 12.7 |
| R412 | SCC | IB2 | 33 | TELE+BRACHY+CHEMO | 86 | Alive | HPV16 | 8.3 |
| R413* | SCC | IB2 | 54 | UNTREATED | 0 | Death | HPV31 | 4.9 |
| R415 | ASCC | IIB | 55 | TELE+BRACHY+CHEMO | 64 | Alive | HPV16 | 8.7 |
| R418 | ACC | IIB | 61 | TELE+BRACHY+CHEMO | 3 | Death | HPV16 | 14.0 |
| R420 | SCC | IIB | 46 | TELE+BRACHY+CHEMO | 32 | Alive | HPV59 | 22.7 |
| R421* | SCC | IIIB | 61 | TELE+BRACHY | 4 | Unknown | HPV51 | 0.4 |
| R422 | SCC | IVB | 57 | TELE | 29 | Death | HPV16 | 1.5 |
| R425 | SCC | IB1 | 64 | TELE+BRACHY | 72 | Alive | HPV59 | 5.0 |
| R426 | SCC | IIB | 67 | TELE+BRACHY+CHEMO | 23 | Death | HPV16 | 0.4 |
| R428 | SCC | IB1 | 45 | HT | 61 | Alive | HPV16 | 4.0 |
| R429 | SCC | IB2 | 46 | TELE+BRACHY+CHEMO | 27 | Alive | HPV52 | 7.2 |
| R430 | ACC | IB1 | 64 | HT | 59 | Alive | HPV16 | 8.3 |
| R432* | SCC | IIIB | 57 | TELE | 7 | Unknown | HPV45 | 0.1 |
| R434 | ACC | IB1 | 34 | HT+TELE+BRACHY | 61 | Alive | HPV16 | 7.6 |
| R437 | SCC | IIA | 72 | HT | 65 | Alive | HPV16 | 3.3 |
| R440 | SCC | IB2 | 45 | TELE+BRACHY+CHEMO | 79 | Alive | HPV16 | 15.4 |
| R441 | ACC | IIB | 24 | TELE+BRACHY+CHEMO | 90 | Alive | HPV16 | 15.2 |
| R443 | SCC | IB1 | 34 | HT+TELE+BRACHY | 71 | Alive | HPV16 | 7.1 |
| R444* | SCC | IIB | 71 | UNTREATED | 26 | Death | HPV16 | 1.3 |
| R446 | ACC | IB1 | 43 | HT | 77 | Alive | HPV16 | 5.7 |
| R451 | SCC | IB2 | 36 | TELE+BRACHY | 84 | Alive | HPV45 | 1.1 |
| R455 | SCC | IIA | 67 | TELE+BRACHY | 85 | Alive | HPV16 | 50.0 |
| R457 | SCC | IIIB | 60 | TELE+BRACHY | 15 | Death | HPV31 | 34.4 |
| R458 | SCC | IIB | 76 | TELE+ HT | 64 | Alive | HPV59 | 2.2 |
| R460 | SCC | IIB | 77 | TELE+BRACHY | 29 | Death | HPV16 | 2.6 |
| R462 | SCC | IVB | 76 | PALLIATIVE CARE | 2 | Death | HPV53 | 0.2 |
| R465 | SCC | IIB | 45 | TELE+BRACHY+CHEMO | 64 | Alive | HPV35 | 4.2 |
| R466* | SCC | IB | 89 | UNTREATED | 39 | Death | HPV33 | 1.2 |
| R468 | SCC | IB2 | 62 | TELE+BRACHY | 62 | Alive | HPV33 | 9.5 |
| R469 | SCC | IVB | 60 | PALLIATIVE CARE | 1 | Death | HPV58 | 17.2 |
| R470 | UND | IIB | 51 | TELE+BRACHY+CHEMO | 3 | Death | HPV58 | 3.0 |
| R475 | SCC | IIIB | 73 | TELE+CHEMO | 14 | Death | HPV52 | 2.0 |
| R478 | SCC | IB1 | 46 | TELE+BRACHY | 72 | Alive | HPV16 | 18.6 |
| R481 | SCC | IIB | 77 | TELE | 6 | Death | HPV16 | 579 |
| R482 | SCC | IB2 | 61 | TELE+BRACHY+CHEMO | 81 | Alive | HPV16 | 6.4 |
| R483 | SCC | IVB | 51 | PALLIATIVE CARE | 11 | Death | HPV16 | 5.3 |
| R485 | SCC | IIA | 60 | TELE+BRACHY | 76 | Alive | HPV16 | 0.1 |
| R487 | SCC | IB1 | 73 | TELE+BRACHY | 14 | Death | HPV45 | 6.7 |
| R488 | SCC | IIA | 55 | TELE+BRACHY+CHEMO | 29 | Death | HPV58 | 5.3 |
| Sample^a^ | Histology^b^ | Tumor stage | Age (years) | Treatment^c^ | Follow up (months) | Status^d^ | HPV type | Fold Change^e^ |
| R493 | SCC | IIB | 50 | TELE+BRACHY+CHEMO | 89 | Alive | HPV45 | 6.6 |
| R495 | SCC | IIB | 68 | TELE+BRACHY | 77 | Alive | HPV16 | 0.1 |
| R496 | SCC | IIIB | 52 | TELE+BRACHY+CHEMO | 77 | Alive | HPV16 | 6.8 |
| R503 | SCC | IIB | 35 | TELE+BRACHY+CHEMO | 13 | Death | HPV45 | 20.4 |

a. A total of 134 samples were screened for the expression of *CDKN3* by RT-qPCR, but only 121 patients were included in the survival study. Patients that did not receive treatment (n=8) or lost during the follow-up in the first 10 months (n=5) were not included in the survival analysis (marked with an asterisk).

b. ACC, Adeno-cell carcinoma. SCC, Squamous-cell carcinoma. ASCC, Adenosquamous-cell carcinoma. UND, Undifferentiated.

c. HT, radical hysterectomy. Tele, teletherapy. Brachy, brachytherapy. Chemo, chemotherapy with Cisplatin.

d. Status alive was registered at the last follow up, death was caused by primary tumor of cervical cancer, except the case labeled with a double asterisk (R221), and unknown cases were lost during the follow up study. The cause of death of case labeled with a double asterisk was unknown.

e. Fold change (FC) was calculated with the median values as follows: expression of *CDKN3* in each tumor/expression of *CDKN3* in the control set.
