# Supplementary material for: CDKN3 mRNA as a Biomarker for Survival and Therapeutic Target in Cervical Cancer
Source: PLoS One. 2015 Sep 15;10(9):e0137397. doi: 10.1371/journal.pone.0137397 (PMC4570808; doi:10.1371/journal.pone.0137397)
Supplement: S3 Table — A, The variants a, b, c, e, f, i, and k have been previously reported in other cancer types, whereas the variants cx1–cx6 are reported here for the first time. Variants cx1–cx5 were amplified in nested RT-PCRs from a 1:5 dilution of the reaction for variant a. Variant cx6 was amplified simultaneously and with the same primers used for variant f. B, The cell lines explored were CaSki, HeLa, and SiHa. C, The variant "a" is the wild type (wt) transcript of the CDKN3 gene. (DOCX) [file pone.0137397.s005.docx]

**S3 Table. Frequency of different mRNA variants of *CDKN3* gene.**

| mRNA variant^a^ |  | Frequency (%) | | | | | | |
| --- | --- | --- | --- | --- | --- | --- | --- | --- |
|  |  | Tumors | | |  | Cell lines (n=3)^b^ |  | Normal cervical epitheliums (n=22) |
|  |  | Death patients (n=16) | Alive patients (n=29) | All patients (n=45) |  |  |  |  |
| a^c^ |  | 16 (100) | 29 (100) | 45 (100) |  | 3 (100) |  | 19 (86.3) |
| b |  | 0 (0) | 0 (0) | 0 (0) |  | 0 (0) |  | 0 (0) |
| c |  | 0 (0) | 0 (0) | 0 (0) |  | 0 (0) |  | 0 (0) |
| e |  | 0 (0) | 0 (0) | 0 (0) |  | 0 (0) |  | 0 (0) |
| f |  | 16 (100) | 26 (89.6) | 42 (93.3) |  | 3 (100) |  | 19 (86.4) |
| i |  | 13 (81.3) | 29 (100) | 42 (93.3) |  | 3 (100) |  | 21 (95.5) |
| k |  | 13 (81.3) | 26 (89.6) | 39 (86.7) |  | 3 (100) |  | 15 (68.2) |
| cx1 |  | 0 (0) | 0 (0) | 0 (0) |  | 1 (33.3) |  | 1 (4.5) |
| cx2 |  | 0 (0) | 0 (0) | 0 (0) |  | 1 (33.3) |  | 0 (0) |
| cx3 |  | 0 (0) | 4 (13.8) | 4 (8.9) |  | 0 (0) |  | 2 (9.1) |
| cx4 |  | 5 (31.3) | 4 (13.8) | 9 (20) |  | 0 (0) |  | 1 (4.5) |
| cx5 |  | 1 (6.3) | 8 (27.6) | 9 (20) |  | 0 (0) |  | 3 (13.6) |
| cx6 |  | 14 (93.3) | 24 (88.9) | 38 (90.5) |  | 3 (100) |  | 19 (86.4) |

a. The variants a, b, c, e, f, i, and k have been previously reported in other cancer types, whereas the variants cx1–cx6 are reported here for the first time. Variants cx1–cx5 were amplified in nested RT-PCRs from a 1:5 dilution of the reaction for variant a. Variant cx6 was amplified simultaneously and with the same primers used for variant f.

b. The cell lines explored were CaSki, HeLa, and SiHa.

c. The variant "a" is the wild type (wt) transcript of the *CDKN3* gene.
